# Temporal Expression Profiling Identifies Pathways Mediating Effect of Causal Variant on Phenotype

**DOI:** 10.1371/journal.pgen.1005195

**Published:** 2015-06-03

**Authors:** Saumya Gupta, Aparna Radhakrishnan, Pandu Raharja-Liu, Gen Lin, Lars M. Steinmetz, Julien Gagneur, Himanshu Sinha

**Affiliations:** 1 Department of Biological Sciences, Tata Institute of Fundamental Research, Mumbai, India; 2 Gene Center, Ludwig-Maximilians-Universität, Munich, Germany; 3 European Molecular Biology Laboratory, Genome Biology Unit, Heidelberg, Germany; 4 Department of Genetics, Stanford University School of Medicine, Stanford, California, United States of America; 5 Stanford Genome Technology Center, Stanford University, Palo Alto, California, United States of America; Washington University School of Medicine, UNITED STATES

## Abstract

Even with identification of multiple causal genetic variants for common human diseases, understanding the molecular processes mediating the causal variants’ effect on the disease remains a challenge. This understanding is crucial for the development of therapeutic strategies to prevent and treat disease. While static profiling of gene expression is primarily used to get insights into the biological bases of diseases, it makes differentiating the causative from the correlative effects difficult, as the dynamics of the underlying biological processes are not monitored. Using yeast as a model, we studied genome-wide gene expression dynamics in the presence of a causal variant as the sole genetic determinant, and performed allele-specific functional validation to delineate the causal effects of the genetic variant on the phenotype. Here, we characterized the precise genetic effects of a functional *MKT1* allelic variant in sporulation efficiency variation. A mathematical model describing meiotic landmark events and conditional activation of *MKT1* expression during sporulation specified an early meiotic role of this variant. By analyzing the early meiotic genome-wide transcriptional response, we demonstrate an *MKT1*-dependent role of novel modulators, namely, *RTG1/3*, regulators of mitochondrial retrograde signaling, and *DAL82*, regulator of nitrogen starvation, in additively effecting sporulation efficiency. In the presence of functional *MKT1* allele, better respiration during early sporulation was observed, which was dependent on the mitochondrial retrograde regulator, *RTG3*. Furthermore, our approach showed that *MKT1* contributes to sporulation independent of Puf3, an RNA-binding protein that steady-state transcription profiling studies have suggested to mediate *MKT1*-pleiotropic effects during mitotic growth. These results uncover interesting regulatory links between meiosis and mitochondrial retrograde signaling. In this study, we highlight the advantage of analyzing allele-specific transcriptional dynamics of mediating genes. Applications in higher eukaryotes can be valuable for inferring causal molecular pathways underlying complex dynamic processes, such as development, physiology and disease progression.

## Author Summary

The causal path from a genetic variant to a complex phenotype such as disease progression is often not known. Studying gene expression variation is one approach to identify the mediating genes, however, it is difficult to distinguish causative from correlative genes. This becomes a challenge especially when studying developmental and physiological traits, since they involve dynamic processes contributing to the variation and only single static expression profiling is performed. As a proof of concept, we addressed this challenge here in yeast, by studying genome-wide gene expression in the presence of the causative polymorphism of *MKT1* as the sole genetic variant, during the time phase when it contributes to sporulation efficiency variation. Our analysis during early sporulation identified mitochondrial retrograde signaling and nitrogen starvation as novel regulators, acting additively to regulate sporulation efficiency. Furthermore, we showed that *PUF3*, a known interactor of *MKT1* had an independent role in sporulation. Our results highlight the role of differential mitochondrial signaling for efficient meiosis, providing insights into the factors regulating infertility. In addition, our study has implications for characterizing the molecular effects of causal genetic variants on dynamic biological processes during development and disease progression.

## Introduction

Identifying the causative genetic variants associated with complex human diseases is only the first step [1]. The major challenge is to understand how these genetic variants cause the disease. The mediating molecular pathways connecting these variants to phenotypes have been more systematically understood in model organisms than in humans [2]. However, even in model organisms there are several examples where a causal genetic variant is not a component of the annotated pathways associated to a trait, making it difficult to fully understand its molecular basis [3]. Having this complete knowledge for complex diseases has a huge potential for development and evaluation of available therapeutic and preventive strategies to counter these diseases [4].

Studying gene expression variation is a standard approach for identification of the causal path from a genetic variant to disease [5,6]. Many of these causal genetic variants have been resolved to single nucleotide polymorphisms (SNPs). Several studies in multiple organisms have been performed to study the effects of these variants called as expression quantitative trait loci (eQTLs) [7,8]. However, for making predictions for the molecular mechanisms underlying a disease, *trans*-acting SNPs are more challenging than *cis*-acting. This is due to the difficulty in distinguishing causative effects of these SNPs from the correlative effects since a SNP can: i) either affect gene expression and the phenotype independently, or ii) modulate gene expression of downstream molecular players, which in turn causes phenotypic variation (causal mediators), or iii) modulate the phenotype which then affects the gene-expression [5]. A few pragmatic approaches have been recently tested in model organisms to identify the causal mediators by studying gene expression changes. One approach, for instance, involved utilizing expression information for the causal genetic variants from multiple environments, which was a better predictor to identify the causal molecular intermediates by the fact that they interact persistently with the variant [9]. For developmental and physiological processes, gene expression follows complex dynamic patterns [10] and so the effect of eQTLs on gene expression can be highly context-sensitive, depending on the developmental stage, physiological phase or tissue type [11–13]. Therefore, when the causative molecular effects of a genetic variant are being studied by measuring gene expression, knowledge of the particular temporal phase when the causal variant transduces its molecular effects is crucial.

Allele replacement strains have been used extensively for fine-mapping the effects of causal genetic variants associated with a trait [14]. Studying allele-specific gene expression could be yet another useful approach which could be exploited in model organisms such as yeast, to study the precise molecular effects of the causal variant on the trait. This can be done by performing genome-wide expression profiling in a pair of allele replacement strains having the same genetic background except for the allele. Using allele replacement strains, *MKT1(89G)* was identified as a causal genetic variant for an efficient completion of sporulation in yeast, called its sporulation efficiency [15]. *MKT1* is a putative endonuclease and its molecular role is beginning to be, but not completely understood [9,16]. *MKT1* has been mapped as a causative gene for several stress-related complex phenotypes, highlighting its extensive pleiotropy [9,17–22], but its functional role in sporulation remains unclear. The developmental process of sporulation in yeast encompasses two meiotic divisions followed by spore formation [23,24]. A study performed parallel phenotyping analysis for the yeast deletion collection and identified around 200 genes required for optimal sporulation efficiency [25]. These genes are both sporulation-specific (*i*.*e*., required only during meiotic processes) and majorly sporulation-associated (*i*.*e*., required for general cellular functions during sporulation such as nutrient metabolism and respiration). However, the study did not identify *MKT1* as one of these genes. It is also not known if *MKT1(89G)* affects any of these 200 genes or any other gene to increase sporulation efficiency. The first association of *MKT1* and sporulation process was reported in the linkage mapping study between segregants of SK1 and S288c strains [15]. Moreover, *MKT1(89G)* was mapped for sporulation efficiency, the end-point of sporulation process. We do not know at which temporal phase during the course of sporulation (early entry into meiosis, middle progression through meiotic phases, or late spore wall formation), *MKT1* affects meiosis.

In this study, we hypothesized that the use of allele replacement strains for studying genome-wide gene-expression during the temporal phase when the causal variant contributes to the phenotype could provide useful insights for identifying the causal molecular mediators underlying complex trait variation. In a pair of allele replacement strains differing solely for *MKT1* causal allele, we characterized the molecular role of *MKT1(89G)* in yeast sporulation efficiency variation. Using genetic assays and mathematical modeling for the meiotic events, we identified the role of *MKT1(89G)* in the early phases of sporulation. In the specific context of *MKT1(89G)*, we studied the genome-wide transcriptional response particularly in the early phase of sporulation and then genetically tested the candidate mediators. Using such an approach, we identified and confirmed novel pathways mediating the effects of *MKT1(89G)* in sporulation efficiency variation. The molecular findings resulting from our study demonstrate the advantage of studying allele-specific temporal gene expression dynamics to identify the causal pathways linking genetic variant to complex traits.

## Results

### Early effects of causal variant on phenotypic variation

**Fig 1:**
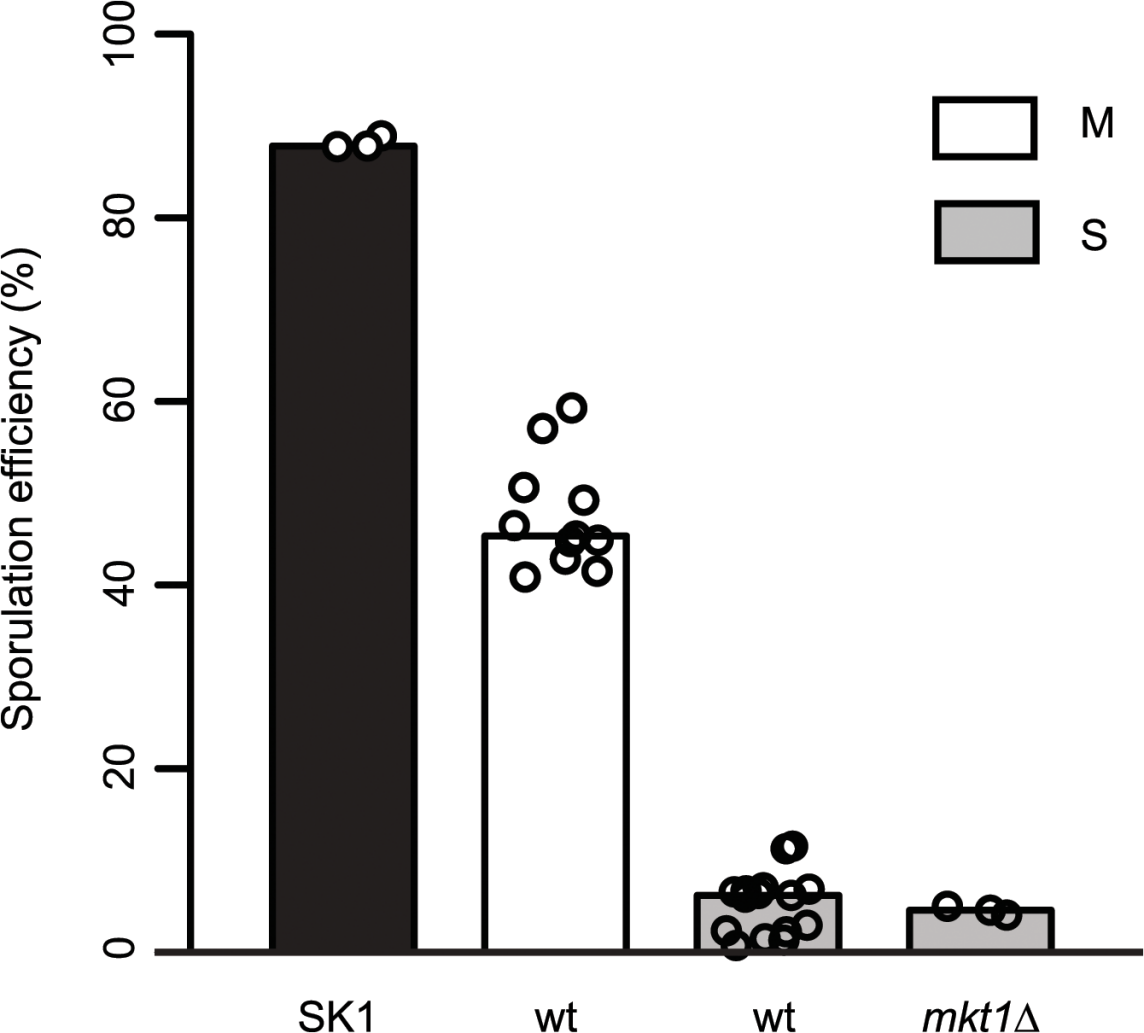
Role of *MKT1* allele in sporulation efficiency variation. Bar plots represent the mean sporulation efficiency, after 48h, of the SK1, M and S strains and S strain with *mkt1*∆. wt indicates wild type strain. The sporulation efficiency data is indicated as circles. A pair test using logit link function was performed (see Methods).

Allele replacement of *MKT1* in the S288c strain from the endogenous adenine (89A) to guanine (89G), of SK1 strain, resulted in increased sporulation efficiency [15]. Whole-genome re-sequencing of the *MKT1* allele replacement strain followed by a series of backcrosses (Methods), was done to confirm that *MKT1(A89G)* was the only sequence difference between the S288c parent (*MKT1(89A)* indicated as “S strain”) and the allele replacement strain (*MKT1(89G)* indicated as “M strain”), the two strains used in this study. After 48h, the high sporulating SK1 strain and the M strain showed increased sporulation efficiency compared to the S strain, which was consistent with the previous report [15] (Fig 1, Table 1, Methods). Compared to the S strain, the SK1 and M strains showed a 17- and a 9-fold increase, respectively (*P* = 1.9x10^-28^, *P* = 1.0x10^-25^, respectively, pair test in Methods). Deletion of *MKT1* in the S strain resulted in sporulation efficiency similar to the S strain, showing that *MKT1(89A)* is a loss-of-function allele for its function in sporulation (Fig 1, Table 1). However, it is possible that the *MKT1(89A)* gene product may have an activity for other phenotypes.

**Table 1:** Mean sporulation efficiency in percentages, with standard deviation (S.D.) of the strains after 48h.

Strain	Mean ± S.D.
SK1	88.2 ± 0.7
S (*MKT1(89A)*)	5.3 ± 3.4
S (*mkt1*∆)	4.5 ± 0.5
S (*puf3*∆)	1.3 ± 1.5
S (*rtg1*∆)	6.7 ± 0.7
S (*rtg3*∆)	5.8 ± 1.0
S (*dal82*∆)	4.4 ± 0.5
M (*MKT1(89G)*)	47.5 ± 6.0
M (*puf3*∆)	28.8 ± 5.8
M (*rtg1*∆)	20.3 ± 1.8
M (*rtg3*∆)	24.8 ± 1.8
M (*dal82*∆)	29.1 ± 4.7
M (*puf3*∆ *rtg3*∆)	14.5 ± 1.5
M (*puf3∆ dal82*∆)	23.9 ± 2.2
M (*rtg3*∆ *dal82*∆)	17.4 ± 1.4

Gene deletions are indicated in brackets. Raw values are given in S2 File.

**Fig 2:**
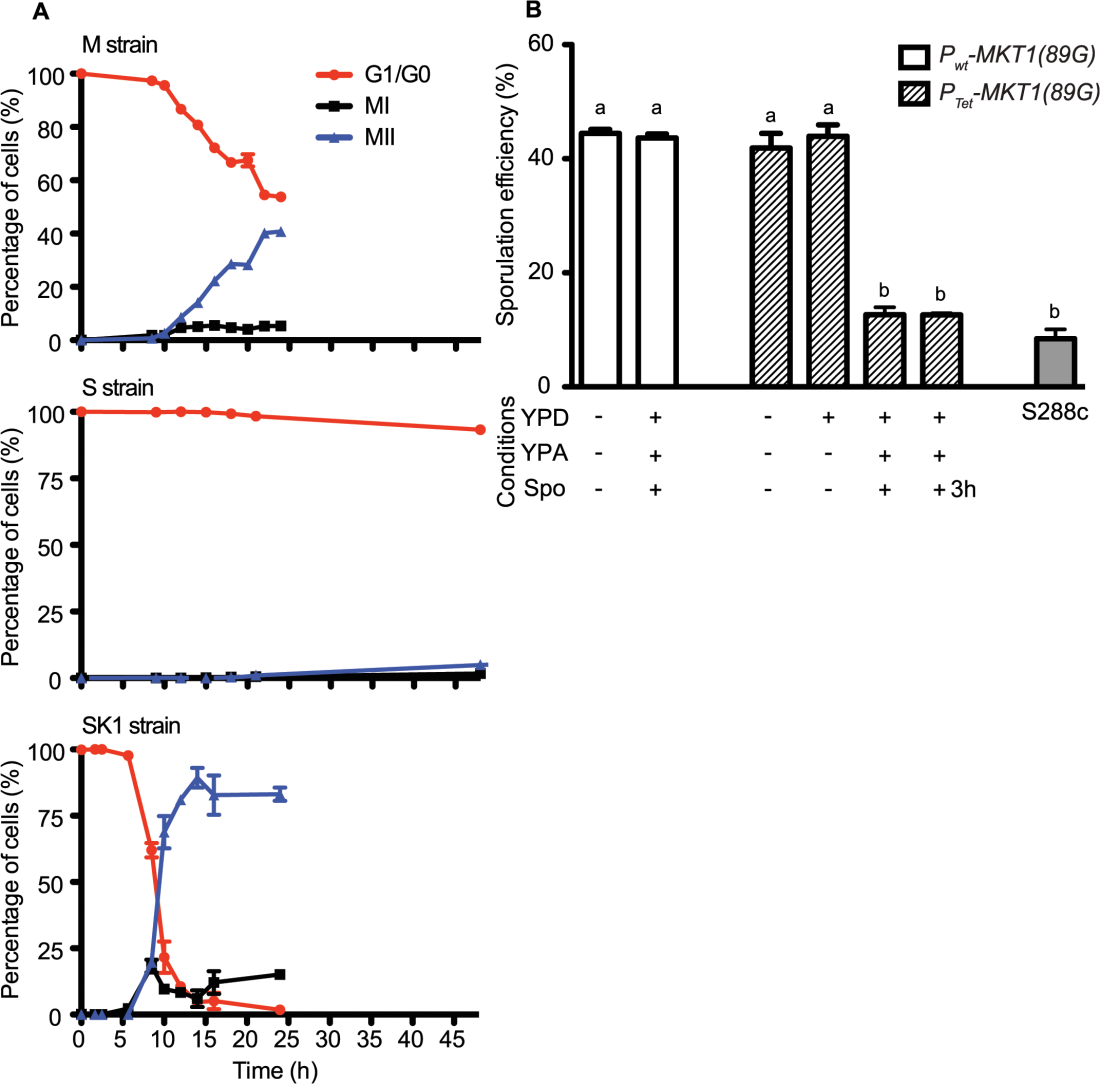
Conditional expression of *MKT1(89G)* during the early phase in sporulation affects the sporulation efficiency. (A) Percentage of 1-, 2- and 4-nuclei states of the M, S and SK1 strains on y-axis. 1-nucleus stage is indicated in red (G1/G2 phase), 2-nuclei state is black (completion of MI phase) and blue is 4-nuclei stage (completion of MII phase). x-axis represents time in sporulation medium. (B) Each strain was grown sequentially in rich (YPD) and pre-sporulation medium (YPA) before incubating in sporulation medium (Spo) for 48h after which sporulation efficiency was estimated. Bar plot represent the mean sporulation efficiency after 48h. *MKT1* expression was switched OFF by addition of doxycycline (indicated as +), and its expression was ON without doxycycline (indicated as-). “+3h” condition indicates that doxycycline was present in the Spo medium for first 3 hours of incubation and was removed from the medium afterwards. Tukey’s multiple comparisons test (*P* < 0.05), bars with the same letter code do not differ significantly. Error bars are the standard errors of mean.

To define the temporal phase during sporulation when *MKT1(89G)* contributes to sporulation efficiency, firstly the proportion of yeast cells completing Meiosis I and II (MI and MII) in the S, M and SK1 strains were quantified (Fig 2A, Methods). M strain started entering MI/II within 10h in sporulation medium, while S strain did not enter MI/II even after 48h. Using these data, multi-stage modeling for the M strain and the parent strains S and SK1 was done to study the distribution of the cell population in different stages of meiosis (Methods, S1 File). As expected, the model predicted that the difference between the M and the S strains occurred during entry into meiosis (initial lag phase of sporulation, S1 Fig). Hence, our observations and the model suggested an early role of the causal variant of *MKT1* in sporulation, which was in agreement with a recent study that showed the contribution of causal variants in critical decision-making steps in the early stages of a phenotypic process [26]. In order to confirm this early role of *MKT1(89G)* in sporulation efficiency variation, a tetracycline-repressible dual-system was used to conditionally express *MKT1(89G)* (Methods). *MKT1(89G)* expression was switched off until 3h after initiation of sporulation, which led to a reduction in the sporulation efficiency of the M strain (P_Tet_-*MKT1*) equivalent to the S strain (Fig 2B, S2 Fig). This result showed that activity of *MKT1(89G)* allele was essential within the first 3h of sporulation.

Meiotic initiation is regulated by multiple nutrient signaling pathways [27]. The functional allele of *MKT1* has a fitness advantage during growth in glucose-rich conditions [9]. Therefore, we tested if increased sporulation efficiency of the M strain is influenced by expression of *MKT1(89G)* during the rich growth medium stage preceding sporulation (Methods). We observed that switching off *MKT1(89G)* during growth in glucose had no effect on sporulation efficiency of the M strain (Fig 2B). Altogether, these results indicated that the role of *MKT1(89G)* during sporulation was independent of its role during growth in glucose and that the allele played a role in the early response to sporulation.

### Genome-wide gene expression response in the presence of causal variant

To identify the pathways through which the *MKT1(89G)* allele affects early sporulation, we studied the entire range of transcriptional response in the S and M strains during the first 10h of sporulation, with denser sampling in the early phase of sporulation (Methods). An extensive remodeling of gene expression was observed in both strains, which increased as time progressed through sporulation (S5 Fig). As expected, the genes involved in sporulation showed a higher expression in the M strain than in the S strain (*P* = 2.0 x 10^–37^, permutation *P* = 0.16, Methods, S6 Fig). Amongst all genes, we identified 862 gene transcripts showing a statistically significant (10% FDR, Methods) differential expression as a function of time between the M and S strains. No enrichment of any functional category within these differentially expressed genes was observed, indicating the pleiotropic role of *MKT1(89G)* and that it might be affecting various aspects of the sporulation process. Comparison of expression profiles of the few known meiotic regulators in the M and S strains showed that *IME1*, the master regulator of meiosis [28], was not differentially expressed. However, *NDT80*, the other crucial regulator of meiosis, involved in meiotic commitment [29], was differentially expressed (S7 Fig, S4 Table). These results suggested that *MKT1(89G)* could affect sporulation at the post-transcriptional level of *IME1* or at the transcriptional level of *NDT80*, both of which could have early regulatory consequences during meiosis [30]. This observation also suggested that the role of *MKT1(89G)* during sporulation might be early and upstream to the regulators of meiosis, in agreement with our earlier results (see Fig 2A and 2B).

**Fig 3:**
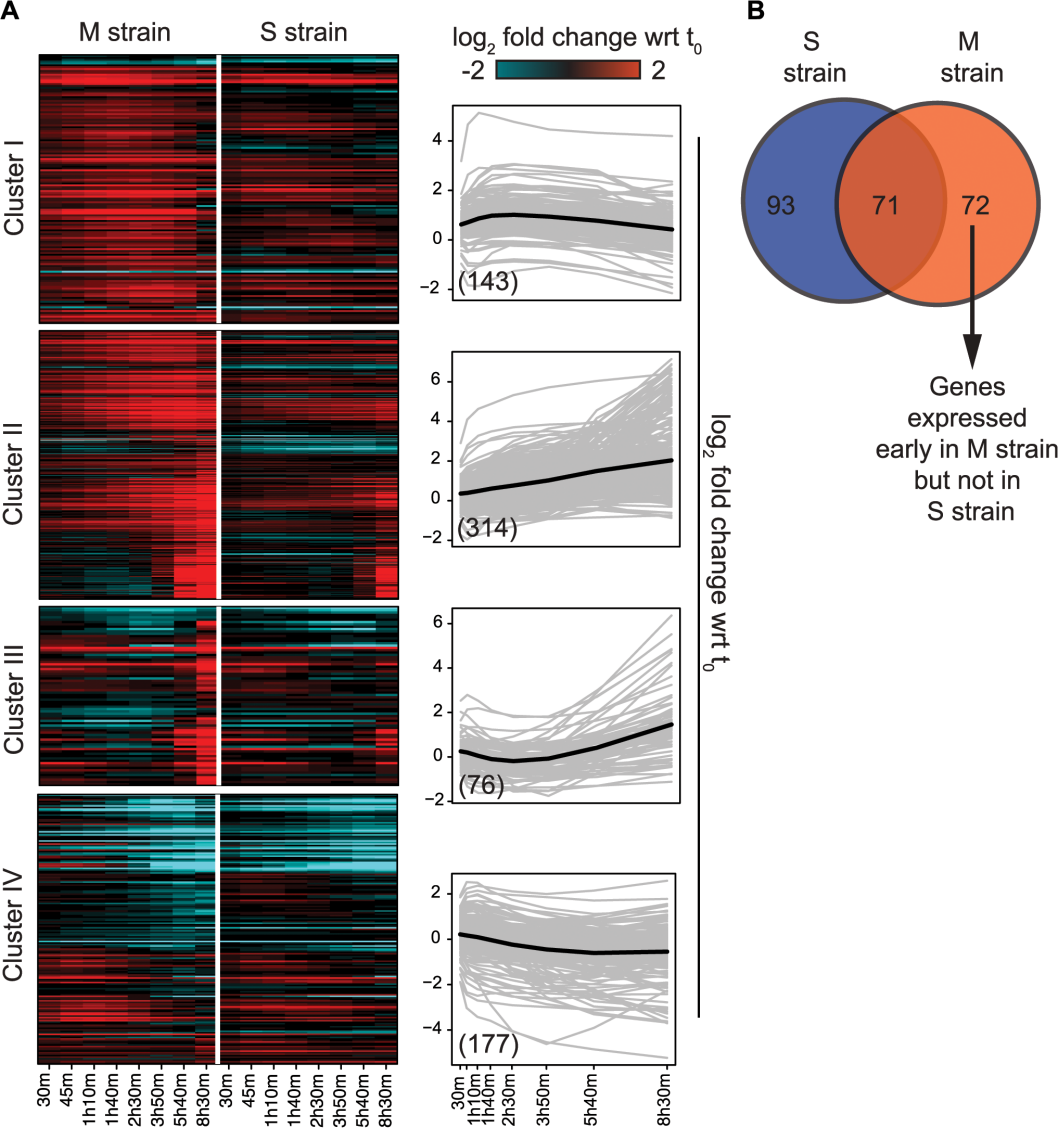
Variation in the gene expression in the presence of *MKT1(89G)* during sporulation. (A) Heat map of the M and S strains showing differentially expressed genes across time within each cluster. The order of genes in the two strains is based on the clustering of the M strain. The average expression profile (black line) of each cluster in the M strain is shown alongside the heatmap. Gray lines show the expression profile for each gene in the cluster. In brackets is the number of genes in each cluster in the M strain. (B) Overlap of early expressing genes of Cluster I between the M and S strains.

**Fig 4:**
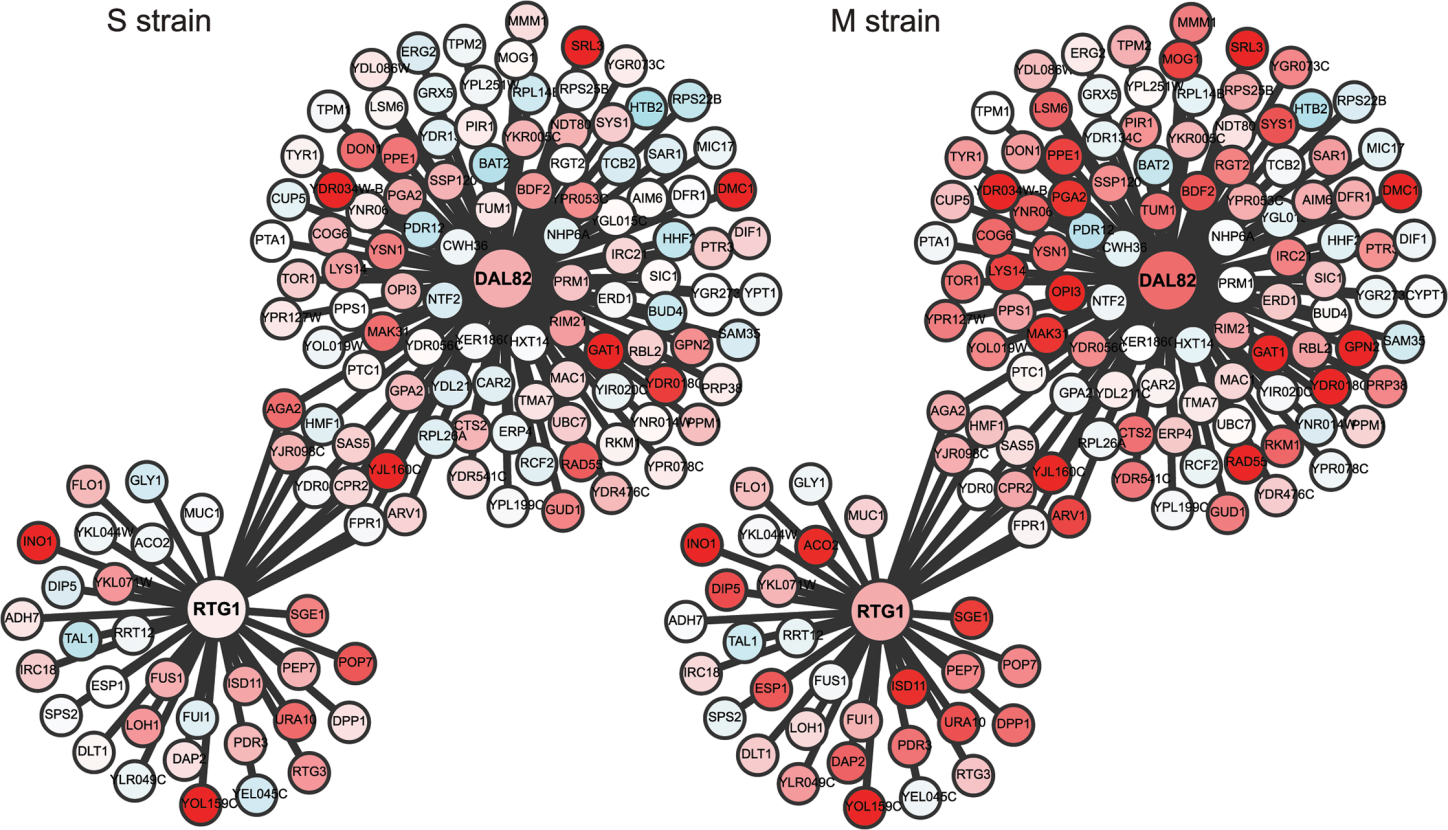
Comparison of the regulatory sub-networks of the transcription factors: Rtg1 and Dal82 in the M and S strains. In each subnetwork, differentially expressed target genes of Rtg1 and Dal82 are shown as nodes connected to their respective regulator. Red color indicates 2-fold overexpression and blue 2-fold repression, calculated as an average of the first three time points in sporulation (early phase). See S8 Table for expression values.

To capture the early role of *MKT1(89G)* during sporulation, genes upregulated early in the M strain and either downregulated or expressed later in the S strain, were considered. Thus, differentially expressed genes were clustered based on their expression profiles, separately for the M and S strains (Methods). Clustering gave six and seven clusters in the M and S strains, respectively, from which four major clusters were identified in each strain (Fig 3A, S5 Table). Clusters I and II consisted of genes mostly expressed in the early stages of meiosis with an enrichment for the target genes of *IME1* and *NDT80*, respectively. In particular Cluster I contained some of the earliest expression changes in the M strain. Comparison of this early cluster between the M and the S strains showed that while 46% (71/143) of its genes overlapped (Fig 3B, S5 Table), the remaining 72 early expressing genes were uniquely differentially expressed in the M strain (S6 Table). We posited that transcription factor(s) whose target genes were significantly enriched within these unique 72 early expressing genes of the M strain might be involved in regulating entry into meiosis. Forty one such transcription factors (*P* ≤ 0.05, odds ratio ≥ 1.5) were identified, which consisted of the regulators of metabolic and mitochondrial signaling (Methods, S7 Table), including sporulation-specific genes, such as *IME1*, *SIN3* and *WTM2* (a *UME1* paralog). To evaluate if the approach we used indeed identified the causal mediating genes contributing to sporulation efficiency variation in the context of *MKT1(89G)*, we selected a few candidate genes from this list of regulators for further investigation. One of the major concerns while studying gene expression is that transcriptional changes can be buffered at the level of phenotype and so do not always manifest themselves in phenotypic variation [31]. Hence, to avoid this buffering while identifying causal regulators of sporulation downstream *MKT1(89G)*, a comprehensive literature survey was done for the selected 41 transcription factors to identify the prime candidate regulators. We did not consider those genes, which have been previously shown to have a causal relationship with sporulation efficiency variation [25]. While prioritizing candidate genes, specifically those regulators were chosen whose functional annotations were related to the processes associated with early regulation of sporulation, such as mitochondrial function and nutrient starvation, but a causal role in sporulation was not known [24,27,32–35]. From this list, *RTG1*, a regulator of mitochondrial retrograde signaling [36] and *DAL82*, a regulator of nitrogen metabolism [37] (Fig 4, S8–10 Figs, S8 Table) were selected for further investigation.

### Identification of novel pathways mediating the causal effects of genetic variant on phenotypic variation

**Fig 5:**
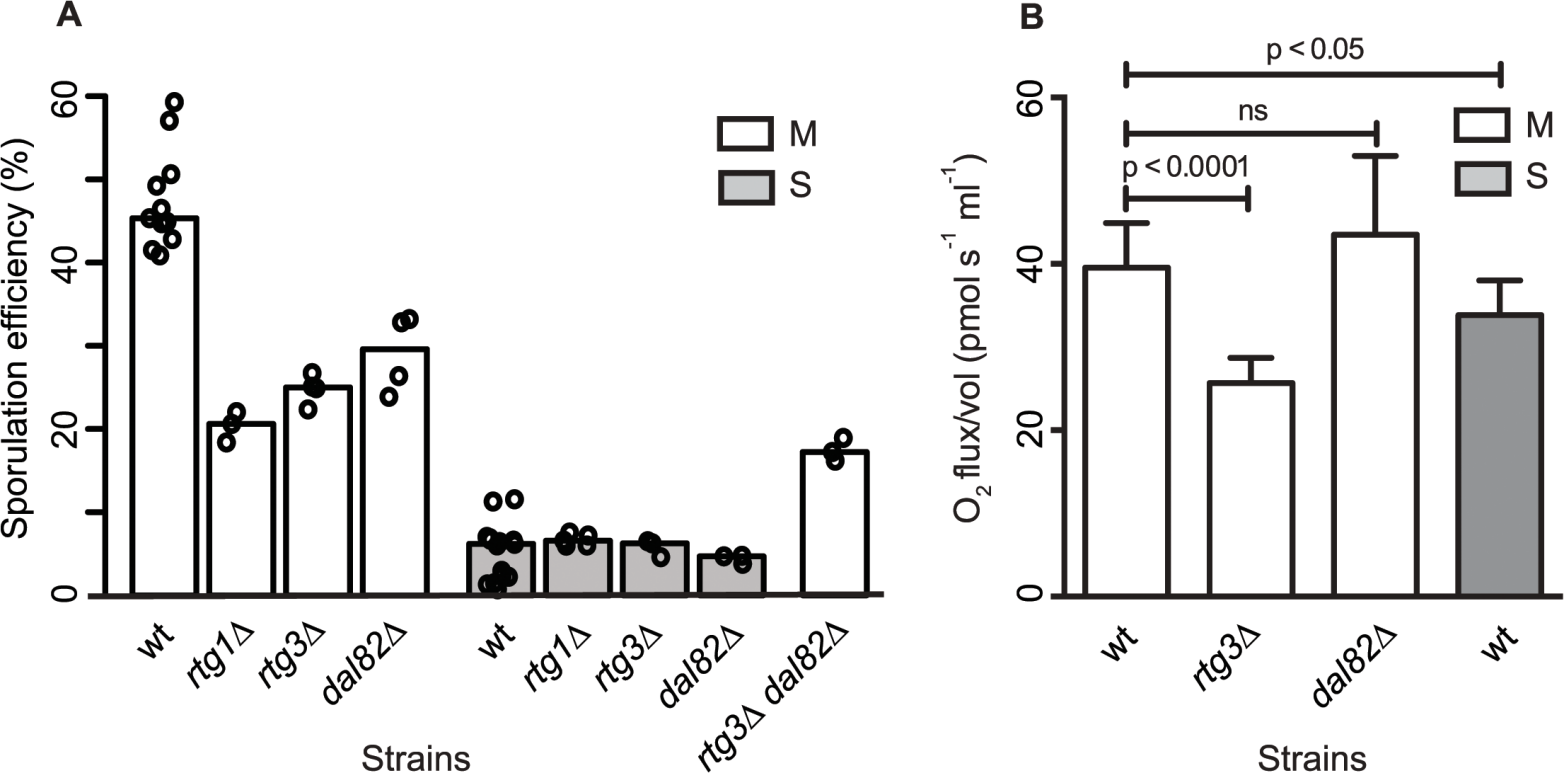
Retrograde signaling and nitrogen starvation regulators mediate the sporulation efficiency variation in *MKT1(89G)*-dependent manner. (A) Bar plots represent the mean sporulation efficiency, after 48h, of single gene deletions of *rtg1*∆, *rtg3*∆, *dal82*∆ both in M and S strains and double deletion of *rtg3∆ dal82∆* in M strain. wt indicates wild type strain. The sporulation efficiency data is indicated as circles. A pair and an interaction test using logit link function were performed (see Methods). (B) Comparison of respiration for the yeast cells incubated for 1h in sporulation medium in the wild type M and S strains, and *rtg3∆* and *dal82∆* in the M strain. Y-axis denotes O_2_ flux/vol (pmol/(s.ml)). *P* was calculated by an unpaired t-test. Error bars are the standard errors of mean.

To test the role of *RTG1* and *DAL82* in sporulation efficiency variation, their deletions in both M and S strains were phenotyped. Another regulator of retrograde signaling *RTG3* [38], a physical interactor and target gene of *RTG1*, showing differential expression in our data, was also deleted in the two strains. Deleting *RTG1*, *RTG3* or *DAL82* reduced the mean sporulation efficiency in the M strain significantly, by about two-fold (*P* = 6.2x10^-10^, *P* = 2.8x10^-10^, *P* = 1.6x10^-7^ respectively, Fig 5A, Table 1, pair test in Methods). This effect was specific to the M strain, because deletion of these genes in the S strain did not affect their mean sporulation efficiency (Fig 5A, Table 1, pair test in Methods); and for *RTG1* and *RTG3*, significant interaction terms were found between the backgrounds (S and M strains) and the deletion for these genes (*P* = 5.8x10^-5^, *P* = 4.7x10^-3^ respectively, interaction test in Methods). *RTG1*, *RTG3* and *DAL82* have not been previously identified as involved in sporulation efficiency as determined from a genome-wide deletion screen [25]. Since this deletion collection was made in the S288c background, carrying the non-functional allele *MKT1(89A)*, this could be a possible reason for the lack of functional implication. A deletion study in the SK1 strain that contains the functional *MKT1(89G)* allele, did not investigate the association of these early sporulation regulators with the process [39]. However, interestingly, an up-regulation of *RTG1* in the early phase of sporulation has been observed in SK1 [40]. These results, thus, support our approach of studying the early effects of the causative allele and implicate novel roles for *RTG1*, *RTG3* and *DAL82* in the early phase of sporulation efficiency downstream to *MKT1(89G)*.

To further investigate if *RTG1/3* and *DAL82* belonged to the same pathway (epistatic effect) or were in separate pathways (additive effect), double deletions for *RTG3* and *DAL82* were phenotyped in the M strain. Deletion of *RTG3* and *DAL82* together reduced the mean sporulation efficiency of the M strain by approximately 3-fold (Fig 5A, Table 1). A non-significant interaction term was obtained between *RTG3* and *DAL82* (interaction test in Methods), indicating that they regulated sporulation efficiency additively, downstream to *MKT1(89G)*. Furthermore, because deletion of *RTG3* and *DAL82* in the M background only partially reduced the sporulation efficiency to that of the S strain (*P* [M (*rtg3∆ dal82∆*) vs. S] = 2.5x10^-7^, Fig 5A, pair test in Methods), these results indicated that these genes explained a partial role of *MKT1(89G)*, and additional complementary pathways were at play.

The mitochondrial retrograde signaling pathway gets upregulated in response to altered mitochondrial function and nutrient starvation. This pathway fine-tunes the metabolic and stress response pathways of the cell by affecting glutamate synthesis and mitochondrial DNA maintenance [33,41]. Since mitochondrial function with regard to respiration is implicated as a critical regulator of sporulation [42], we speculated if differential mitochondrial activity was involved in sporulation efficiency variation in the presence of *MKT1(89G)*. We evaluated the mitochondrial function in the M and S strains by assaying oxygen consumption flux during early sporulation (Methods). The M strain showed a better mitochondrial function than the S strain (Fig 5B) at 1h in sporulation. Deletion of *RTG3* in the M strain decreased this oxygen consumption flux, though *dal82*∆ had no effect on the flux (Fig 5B). These results suggested a role of differential mitochondrial function in sporulation efficiency variation. However, a better understanding of the role of mitochondrial retrograde pathway in sporulation efficiency would require further investigation.

### Role of *PUF3* in sporulation efficiency independent of *MKT1(89G)*

**Fig 6:**
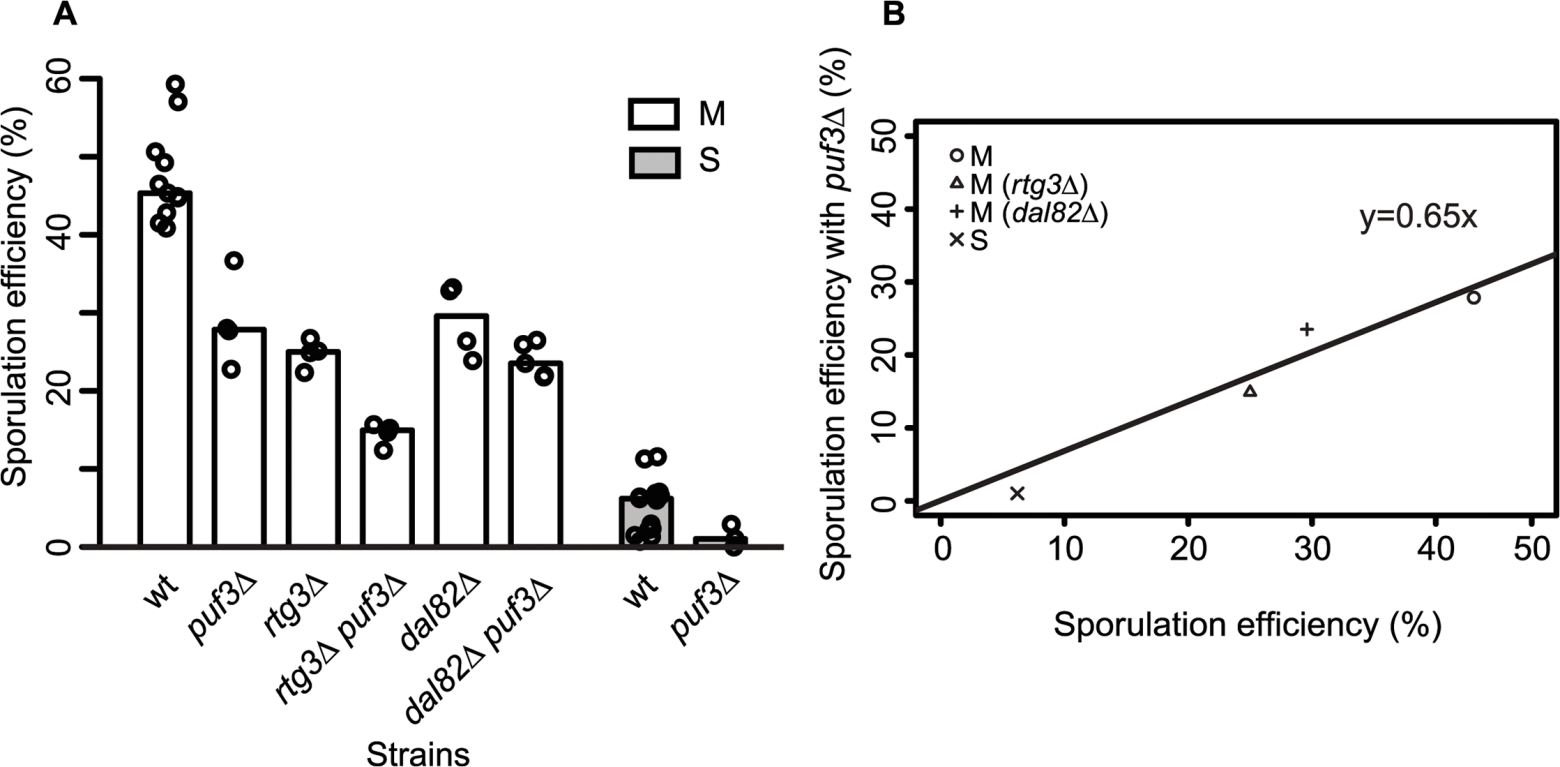
Role of *PUF3* in sporulation efficiency is independent of *MKT1(89G)*. (A) Bar plots represent the mean sporulation efficiency, after 48h, of single gene deletions of *puf3∆*, *rtg3∆*, *dal82∆* and double gene deletions of *rtg3∆ puf3∆*, *dal82∆ puf3∆* in M strains. Single gene deletion of *puf3∆* in S strain is also shown. wt indicates wild type strain. The sporulation efficiency data is indicated as circles. A pair and an interaction test using logit link function were performed (see Methods). (B) The sporulation efficiency of wild type of M and S strains along with single gene deletions of *rtg3*∆, *dal82*∆ in M strain (x-axis) compared to their sporulation efficiency with *puf3∆* (y-axis).

Differential mitochondrial activity in the presence of *MKT1(89G)* suggests a role for the Mkt1 interactor, Puf3, a Pumilio-family protein, which has been suggested to explain the extensive *MKT1(89G)* pleiotropy during mitotic growth in rich media as well as in stress environments [16,22,43]. Puf3 is an mRNA binding protein that regulates the fate of nearly 200 nuclear-encoded mitochondrial transcripts [44]. Even though we found a few *PUF3* target genes (13/214 genes) differentially expressed during sporulation, none were in the set of unique early expressed transcripts in the M strain (S10 Fig). To further evaluate if *PUF3* had a role in sporulation efficiency variation in the presence of *MKT1(89G)*, we deleted *PUF3* in the S and M strains and M strain with single deletions of *rtg3*∆ and *dal82∆*. If *PUF3* has an independent role in sporulation, reduction in sporulation efficiency by *puf3∆* deletion would be independent of the background (*MKT1*, *RTG3* or *DAL82*), and we would observe an additive effect on sporulation efficiency. Any observed significant deviation from this expectation would imply dependence. One extreme case of dependence would be epistasis. In that case, deleting *PUF3* in these backgrounds would not lead to decreased sporulation efficiency. We observed that *PUF3* deletions in all the four backgrounds: M, S, M (*rtg3*∆) and M (*dal82*∆) reduced their sporulation efficiency (regression line y = 0.65x showing around 35% less sporulation efficiency for all strains, Fig 6A and 6B, Table 1, pair test in Methods). Furthermore, interaction terms (Methods) were non-significant for deletion of *PUF3* between the M and the S strains (*P* = 0.49), the M and M (*rtg3*∆) strains (*P* = 0.53), and only mildly significant between the M and M (*dal82*∆) strains (*P* = 0.02). These results indicated that the effect of *PUF3* on sporulation efficiency was independent of *MKT1(89G)* and its downstream genes *RTG1/3* and *DAL82*.

## Discussion

**Fig 7:**
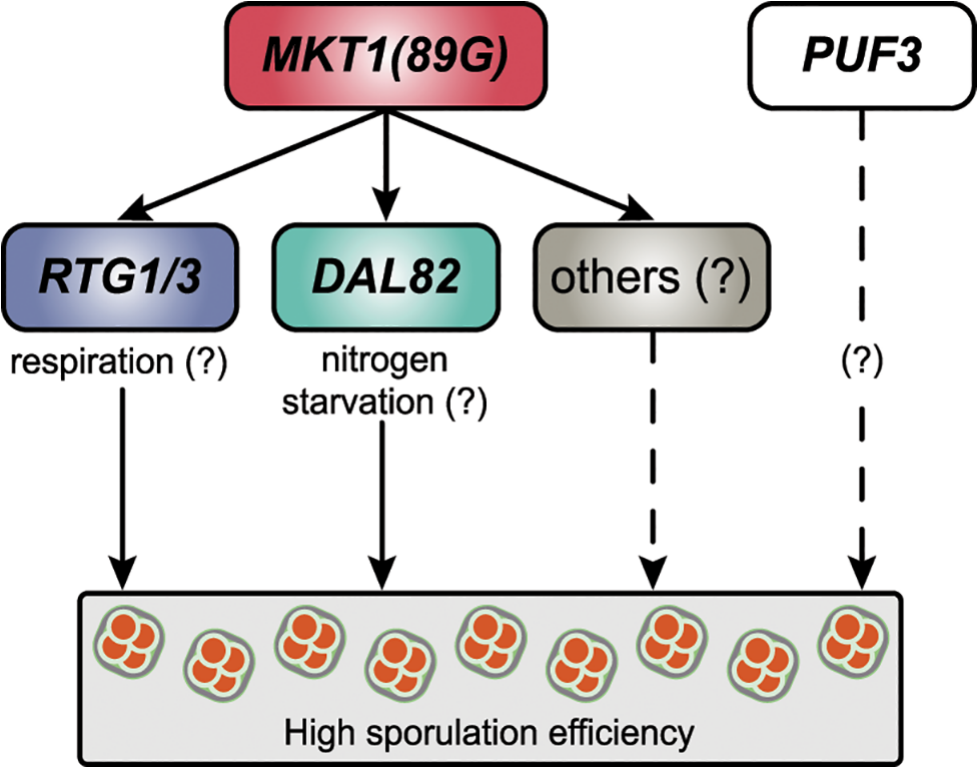
A model for the molecular basis of the *MKT1* causal variant in the sporulation efficiency variation. *MKT1(89G)* genetically interacts with *RTG1/3* and *DAL82* (solid lines) to possibly modulate respiration and nitrogen metabolism in order to increase sporulation efficiency. *MKT1(89G)* also interacts (dashed lines) with other molecular pathways to further affect sporulation efficiency. *PUF3* shows a *MKT1(89G)*-independent role (dashed lines) in sporulation efficiency variation.

Over the past decade a detailed genotype-phenotype map for complex traits including diseases has been determined [45], however, a functional map defining how causal genetic variants (alleles) modulate the underlying pathways resulting in phenotypic variation, is missing. Filling this functional gap will help to identify molecular candidates for therapeutic intervention in human diseases and to make useful predictions regarding response to a particular therapy and survival of a patient [1]. The first step to characterize this functional genotype-phenotype map requires identification of the causal mediating genes in a biological network regulating the phenotype. Investigation of the intermediate phenotypes *viz*. transcripts, proteins and metabolites, is routinely used to identify these causal mediators [46]. In this study we demonstrate a couple of steps essential for accurate identification of these causal molecular mediators: i) studying allele-specific temporal dynamics of the biological processes underlying complex traits, and ii) allele-specific functional validation of the predicted mediators. We report the characterization of molecular pathways modulated by a causal genetic variant in a dynamic biological process using the above approach. In particular, we studied the molecular effects of the essential *MKT1(89G)* allele on the yeast transcriptome during sporulation. We not only identified novel pathways regulating the phenotype, but also confirmed the independent role of a known interactor (Puf3) of *MKT1(89G)* in the phenotype (Fig 7). *MKT1(89A)* is not a naturally occurring allele, observed only in the S288c strain [20]. However, such rare polymorphisms are receiving increasing attention for their contribution to common human diseases [47]. In this sense, our approach has a general applicability since it can be applied to study the molecular basis of both common and rare variants.

Using our approach of studying early gene expression dynamics in response to the *MKT1(89G)* allele, we identified that regulators of mitochondrial retrograde signaling and of nitrogen starvation act additively to regulate sporulation efficiency (Fig 5A). Mitochondria responds to a wide array of stresses by inducing various complex cellular responses and promoting cellular adaptation to reduce the impact of further stressors [48]. Mitochondrial retrograde signaling is one of the stress signaling responses of the cell during mitochondrial functional alteration and glutamate starvation [33]. It affects mitochondrial DNA maintenance [49] and hence the respiratory competency of a cell. During meiosis in yeast cells, energy production occurs through the Krebs cycle [32,35,42], and hence respiration is a critical regulator of meiosis in yeast [42] and in humans. In humans, low mitochondrial DNA has been associated with ovarian insufficiency [50]. We observed an improved mitochondrial activity during early sporulation in the M strain compared to the S strain (Fig 5B). A reduction in this high mitochondrial activity in the absence of mitochondrial retrograde signaling regulator *RTG3* indicated that *MKT1(89G)* might confer a better stress response through *RTG3*, with increased sporulation efficiency being one of the consequences. This role of retrograde signaling in regulation of developmental processes responding to nutritional stresses has been shown for pseudohyphal growth in yeast [51]. Further investigating this association of differential mitochondrial signaling, particularly retrograde signaling with meiosis and development in general can help provide insights into the factors regulating infertility.

In this study, we characterized the essential role of *MKT1(89G)* allele in sporulation efficiency. This allele was particularly interesting to study as this coding polymorphism of *MKT1* is present in all laboratory strains (except strains isogenic to S288c), as well as clinical and natural isolates of yeast including the SGRP strain collection [15,18,20,52]. Since the previous genetic screens [39,25,53] or genome-wide expression studies [40,54] for sporulation and sporulation efficiency, were done in the S288c background carrying the *MKT1* allele which is non-functional in sporulation, this could be a possible reason for not identifying *MKT1* to be involved in the process. The founder strain of S288c, EM93 carries the *MKT1(89G)* allele suggesting that during domestication of S288c this functional allele was lost [20,55]. During evolution of S288c in low-glucose conditions, the native *MKT1(89A)* mutated to *MKT1(89G)* within 500 generations [56], also indicating the crucial role of *MKT1(89G)* in stress-related conditions. Altogether, these observations demonstrate the limitations of studying genotype-phenotype relationships in a single genetic background, especially in laboratory strains, which might have degenerated their stress response machinery partially or completely, as a result of domestication [57].

Using our approach, we further showed an *MKT1(89G)*-independent role of *PUF3* in meiosis (Fig 6A and 6B). This was surprising since eQTL mapping studies have suggested *MKT1* as a global regulator of gene expression [22,58] and have identified its most upstream interactors, such as *PUF3*, during mitotic growth in multiple environments [9,16]. Puf3 regulates translation and degradation of nuclear-encoded mitochondrial mRNAs by localizing them near mitochondria or P-bodies, which are cytoplasmic sites for mRNA decay and stalling [16,44,59,60]. Since *MKT1* has a post-transcriptional regulatory role both in yeast [61] and in trypanosomes [62], its interaction with *PUF3* suggested a probable mechanism for understanding the role of *MKT1*. However, for sporulation efficiency, we observed that Puf3 showed an *MKT1(89G)*-independent role. We, therefore, speculate that Puf3 might be a mitotic growth-specific interactor of *MKT1(89G)*. Its role in sporulation efficiency, though, could involve post-transcriptional regulation of mitochondrial mRNAs through P-bodies during sporulation. In *Drosophila*, *C*. *elegans*, mice and mammals [63,64], P-bodies related RNA granules are known to be involved in translational control of germ cell transcripts. However, in yeast, P-bodies have been observed only during glucose starvation and stress conditions such as ethanol tolerance [22,65]. Therefore, our results indicate an interesting interaction between Puf3 and sporulation efficiency variation and this could be a future line of investigation to determine if P-body formation has a regulatory role in yeast meiosis.

Through our analysis, we attempted to understand the molecular basis of a complex trait. Using an allele-specific approach, we determined and functionally validated the molecular consequences of a single causative variant in phenotypic variation. This approach helped to identify novel associations between mitochondrial and metabolic pathways with meiosis. Further analyses of these expression data can identify additional regulators and pathways involved in sporulation efficiency variation in the presence of *MKT1(89G)* (Fig 7, S7 Table). This approach demonstrated in yeast can be applied to higher eukaryotes to study transcriptional dynamics of developmental processes or progression of diseases. This will assist in understanding the precise genetic effects of a causal variant, improving the existing genotype-phenotype functional relationship map.

## Materials and Methods

### Yeast strains, growth and sporulation conditions

Whole-genome resequencing of the *MKT1* allele replacement strain (S9 Table) was performed to confirm the presence of the causative SNP (details in S1 Table, S1 Text (Section 1)). Backcrossing the haploid allele replacement strain to the S288c parent strain three consecutive times (details in S1 Text (Section 2)) confirmed that homozygous *MKT1(A89G)* was the only sequence difference between the diploid S288c parent (S strain) and the allele replacement strain (M strain). All the S (*MKT1(89A)*) and M (*MKT1(89G)*) strains used in this study were derivatives of S288c strain except SK1 strain (S9 Table). The strains were grown at 30°C in YPD (1% yeast extract, 2% bacto peptone, 2% dextrose) and YPA (1% yeast extract, 2% bacto peptone, 1% potassium acetate). Deletions were performed in the haploids by replacing the specific ORF with one of the dominant drug-resistance cassettes (*hphMX4*, *kanMX4* or *natMX4*) which were PCR-amplified from their respective plasmids as described previously [66]. The strains were transformed using the standard lithium acetate-based method [67] and homologous integration of the deletion cassette was confirmed by performing a colony PCR for both the ends. Three confirmed independent transformants were selected to minimize random mutations during the transformation step, diplodized using pHS2 plasmid (containing a functional *HO*) and phenotyped. All further experiments were performed using the diplodized parent strains and their diploid derivatives. The primers for deletions and their confirmations are listed in S10 Table. Sporulation conditions and the calculation of sporulation efficiency was done as previously described [68] in liquid sporulation medium (1% potassium acetate supplemented with 20mg/ml uracil, 20mg/ml histidine, 30mg/ml leucine, 20mg/ml methionine and 30mg/ml lysine). For each strain, minimum three biological replicates were used and approximately 1,000 cells were counted per replicate. Fold difference was calculated as the ratio of mean sporulation efficiencies of the two strains A and B when the sporulation efficiency of A is greater than of B.

### Statistical analysis of sporulation efficiency data

Two statistical tests were used: the pair test and the interaction test. The pair test tests the null hypothesis that two given strains have the same sporulation efficiency. To this end, the number *y*_*i*,*k*_ of sporulated cells (4-nuclei count) among the total number of cells *n*_*i*,*k*_ of strain *i* in replicate experiment *k* was modeled with a quasi-binomial generalized linear model using the logit link function and subject to a common log-odd ratio *β*_*i*_ between replicates, *i*.*e*.:

$$\text{log}\left(\frac{\mu_{i,k}}{n_{i,k}-\mu_{i,k}}\right)=\beta_{i}\;\text{for all}\;k,$$

where *μ*_*i*,*k*_ = *E*(*y*_*i*,*k*_). The pair test tests the null hypothesis of equality of log odd-ratios for two strains *i* and *j*, *i*.*e*. *H*_0_: *β*_*i*_ = *β*_*j*_.

The interaction test tests the null hypothesis that the effect of mutation A is independent of the effect of mutation B, taking the M strain as reference background. This test thus compares four strains: mutation A only, mutation B only, both A and B and neither A nor B (M strain). Here, the strain S was considered as a M strain mutated for *MKT1(89)*. For every interaction test, we considered the dataset of the four strains of interest and fitted a quasi-binomial generalized linear model using the logit link function and subject to:

$$\text{log}\left(\frac{\mu_{i,k}}{n_{i,k}-\mu_{i,k}}\right)=\beta_{0}+\beta_{A}A_{i}+\beta_{B}B_{i}+\beta_{A,B}A_{i}B_{i}\;\text{for all}\;k,$$

where, *A*_*i*_ and *B*_*i*_ are indicator variables of the mutations A and B in strain *i* respectively. The interaction test tested the null hypothesis that the odd ratio of sporulation in the double mutant equals the product of the odd ratios of each mutation, *i*.*e*. *H*_0_: *β*_*A*,*B*_ = 0.

Both the pair test and the interaction test were implemented in the statistical language R with the function *glm()* assuming a constant variance function fitted by maximizing the quasi-likelihood and using the t-test on tested parameters (see S2 File for raw data and R script).

### Estimating the progression through meiotic phases

Aliquots of sporulating cells of M strain culture were fixed with ethanol at regular intervals (as indicated in Fig 2A) from 0 to 48h in the sporulation medium. These time-points were chosen to capture the progression through meiotic stages in the strain. Samples were stained with DAPI (4’-6’ diamidino-2-phenylindole) using the standard methods [69] for calculating the proportion of cells with 1-nucleus (Non-sporulating/G1), 2-nuclei (MI) and 4-nuclei (MII) using Carl Zeiss Axiovert 200 fluorescence microscope. For each strain, proportion of cells were counted till saturation was reached for two consecutive time points. Grey scale images were captured using a CCD camera and pseudo-coloured using the image acquisition software (Axiovision) supplied with the microscope. To estimate the sporulation efficiency and DAPI staining, 1,000 cells from the three biological replicates for each strain were counted.

### Mathematical modeling for progression through meiotic phases

A multi-stage modeling was performed (details and raw data in S1 File). Cells in G1/S phase of cell cycle are said to be in 1-nucleus state. Cells that have completed MI or MII are said to be in 2-nuclei or 4-nuclei state, respectively. Cells that did not progress from one cell cycle state to another are mentioned as inactive cells. The existence of inactive states is supported by the fact that at steady state, some cells still have one nucleus or 2-nuclei indicating they are trapped at these stages, which could be possibly due to nuclear destruction mechanism resulting in dyads [70]. Hence, cells could be either in a 1-nucleus active, 1-nucleus inactive, 2-nuclei active, 2-nuclei inactive or 4-nuclei state. Moreover the cells were assumed to only progress in one direction (no back transitions) from the 1-nucleus active to either the 1-nucleus inactive or the 2-nuclei active stage, and from the 2-nuclei active to either the 2-nuclei inactive or to the 4-nuclei state. The samples contain a large number of cells and thus we used Ordinary Differential Equations to describe the dynamics of the system. The dynamics was modeled with an initial lag phase (measured as *τ*) followed by first order kinetics between the stages (measured as *α*, *β*, *γ* and *δ*, as shown below).

$$\left(\begin{array}{l} X_{1}\overset{\;\alpha \;}{\to }X_{2}\overset{\;\gamma \;}{\to }X_{4}\\ X_{1}\overset{\;\beta \;}{\to }Y_{1}\\ X_{2}\overset{\;\delta \;}{\to }Y_{2} \end{array}\;\right)$$

where, *X*_1_ is proportion of cells in 1-nucleus active stage, *X*_2_ in 2-nuclei active stage, *X*_4_ in 4-nuclei active stage, *Y*_1_ is proportion of cells in 1-nucleus inactive stage, *Y*_2_ in 2-nucleus inactive stage. The model was fitted by minimizing least square errors to the measured proportions of the cells with 1, 2, and 4-nuclei, measured along the time. Confidence intervals were obtained by bootstrap of the data.

### Conditional expression of *MKT1* during sporulation

*tetO*_*7*_-based promoter substitution cassette containing *kanMX4*, amplified from the plasmid pCM225 [71], was inserted to replace the endogenous *MKT1* promoter (-300 to -1bp upstream start site) in the M strain (P_Tet_-*MKT1*). M strains with the endogenous promoter (P_wt_-*MKT1*) and the *tetO*_*7*_ promoter (P_Tet_-*MKT1*) were grown in a glucose-rich medium (YPD) and synchronized in pre-sporulation medium (YPA) prior to initiating sporulation. To determine the concentration of doxycycline at which the effect of *MKT1(89G)* on sporulation efficiency is similar to *MKT1(89A)* (implying *MKT1(89G)* is not functional or OFF), the P_Tet_-*MKT1* strain was grown and sporulated in 2, 3 and 5μg/ml of doxycycline and phenotyped by estimating the sporulation efficiency after 48h. At 5μg/ml doxycycline, the sporulation efficiency of the P_Tet_-*MKT1* strain was similar to the S strain (S2 Fig) and this concentration was used for further experiments. To switch off *MKT1(89G)* expression only during the growth in glucose, the P_Tet_-*MKT1* strain was grown in YPD with doxycycline, washed and added to YPA and the sporulation medium in the absence of doxycycline. For switching off *MKT1(89G)* throughout the sporulation process, doxycycline was added to all the three media (YPD, YPA and sporulation). To switch off *MKT1(89G)* till 3h in sporulation medium, doxycycline was added in YPD, YPA and sporulation medium. Cells were washed after 3h in sporulation and resuspended in the sporulation medium without doxycycline till 48h, and were phenotyped. A complementary experiment where *MKT1(89G)* was switched ON till 3h in sporulation medium and switched OFF from 3h to 48h in sporulation was done by adding doxycycline in sporulation medium post 3h in sporulation medium (S2 Fig). For each strain in each condition, minimum three biological replicates were used and approximately 1,000 cells were counted per replicate per condition for estimation of sporulation efficiency. The means and variances were tested for significance using one-way ANOVA followed by Tukey’s multiple comparisons test (Prism, Graphpad Software Inc.). Statistical significance was determined at *P* < 0.05.

### Transcriptional profiling, normalization, smoothing and baseline transformation

Temporal transcriptome profiling was performed for the sporulating yeast cells at 0h, 30m, 45m, 1h10m, 1h40m, 2h30m, 3h50m, 5h40m and 8h30m (logarithmic time-series) in the sporulation medium. For this, 100ml aliquots of the culture were pelleted and stored at -80°C. Transcriptome profiling was performed using the *S*. *cerevisiae* yeast tiling array (Affymetrix, Cat# 520055) as described previously [72]. Time-series arrays of M and S strains in sporulation were normalized by *vsn* (S1 Text (Section 3), S3 Fig) [73].

Using log_2_ transformed expression values, after normalization (S2 Table), the expression profiles of all transcripts of S and M strains were made continuous over time using *locfit* [74] with the bandwidth parameter ‘*h*’ optimized at 1.21 (S1 Text (Section 4), S4 Fig, S3 Table). A baseline transformation for each transcript, after smoothing, was done by subtracting each time point value from t = 0h (*t*_0_).

$$\begin{array}{c} y{'}_{S(t_{n})}=y_{S(t_{n})}-y_{S(t_{0})}\\ y{'}_{M(t_{n})}=y_{M(t_{n})}-y_{M(t_{0})} \end{array}$$

where, *y* is the expression value of a transcript for a strain (S or M) at a specific time point and *y’* is the transformed expression value.

To compare the sporulation genes (obtained from Deutschbauer *et al*. [25]) between the M and S strains, their expression in the two strains were tested using 1,000 permutations of Wilcoxon test on an equal number of randomly selected genes (S6 Fig). R scripts used for the analyses are given in the S3 File.

### Identification of differentially expressed genes using EDGE

To identify differentially expressed genes (after removing tRNAs, snRNAs and transcripts from terminal repeats) between the two strains, the temporal expression profiles of each transcript was compared using the method implemented in the EDGE (Extraction of Differential Gene Expression) software [75]. One thousand permutations were done to calculate the null distribution with a random number seed. EDGE analysis identified transcripts of 862 significant differentially expressed genes across time (10% FDR, S4 Table). Within these 862 genes, a subset of differentially expressed transcription factors and differentially expressed targets of all the transcription factors (obtained from the YEASTRACT database, [76] were selected. This subset of 727 genes was used for further analysis.

### Clustering of identified genes using TimeClust

The 727 differentially expressed genes were clustered according to their temporal expression patterns using time abstraction method implemented in the TimeClust software [77]. The smoothened and baseline transformed expression data of the 8 sporulation time-points was analysed with window span parameter set at 3. An absolute expression change of 0.1 was considered as a change. This clustering method was applied on the expression data separately for the two strains resulting in six and seven clusters in the M and S strains, respectively (S5 Table). The gene lists of the M and S strains for the Cluster I, consisting of early expressing genes, were compared. For the genes unique to the M strain in this cluster (S6 Table), the transcription factors regulating them were extracted using the YEASTRACT database (S7 Table) [76].

### Estimation of oxygen flux to evaluate mitochondrial function

After 1h in sporulation, 5 x 10^6^ cells from each of the three biological replicates were used for the assay. Oxygen consumption flux was determined, in total volume of 2.1ml sporulation medium at 30°C with 500 rpm, using OROBOROS O2k high-resolution respirometer (OROBOROS Instruments Corp., Innsbruck, Austria). Data acquisition and calculation of oxygen flux was done according to the manufacturer’s instruction in DatLab software. Unpaired Student’s t-test (Prism, Graphpad Software Inc.) was performed for comparing differences between the means of the two strains. Statistical significance was determined at *P* < 0.05.

## Supporting Information

The Supporting information is also available at: http://www.tifr.res.in/~dbs/faculty/hsinha/MKT1Spo

S1 Fig Early role of *MKT1(89G)* in sporulation predicted through modeling. Boxplot showing the initial lag phase (in hours) of the strains (x-axis) in entering meiosis I, calculated by the parameter tau (y-axis). See Methods for details of modeling.
(PDF)

S2 Fig Conditional expression of *MKT1(89G)* during sporulation. Each strain was grown sequentially in rich (YPD) and pre-sporulation medium (YPA) before incubating in sporulation medium (Spo) for 48h after which sporulation efficiency was estimated. Bar plot represent the mean sporulation efficiency after 48h. (A) Testing doxycycline concentration for switching off *MKT1* expression during sporulation. *MKT1* expression was switched OFF in all the three conditions by addition of doxycycline (indicated as +dox). No doxycycline in any of the three media is indicated as-dox (implying *MKT1* expression ON). Concentration of doxycycline is depicted on x-axis. Tukey’s multiple comparisons test (*P* < 0.05) was performed. In both concentrations 3μg/ml and 5μg/ml of doxycycline, M strain showed sporulation efficiency equivalent to S strain. 2μg/ml doxycycline showed significant difference in mean sporulation efficiency compared to S strain. Further experiments were performed using 5μg/ml doxycycline. Error bars are the standard errors of mean. (B) Early role of *MKT1* expression. *MKT1* expression was switched OFF by addition of doxycycline (indicated as +), and *MKT1* expression was ON when no doxycycline was added (indicated as-). “-3h” condition indicates that no doxycycline was added till 3h in sporulation medium. “+3-48h” condition indicates that *MKT1* expression was switched OFF 3h-post initiation of sporulation, by adding doxycycline during 3–48h in sporulation medium. Tukey’s multiple comparisons test (*P* < 0.05), bars with the same letter code do not differ significantly. Error bars are the standard errors of mean.
(PDF)

S3 Fig Normalization of the time-series expression data. The expression for each transcript in the two replicates has been plotted against each other. Replicate 1 is in x-axis and replicate 2 is in y-axis. Red line indicates the normal line expected if there was a 100% correlation between the replicates.
(PDF)

S4 Fig Smoothing of normalized temporal data using *locfit*. Representative images showing normalized (black line) and normalized *locfit* (red line) data in M and S strain. x-axis denotes the time-points in sporulation medium and y-axis is the log_2_ expression.
(PDF)

S5 Fig Scatterplots comparing expression of all the genes in M and S strain across time-point. The expression (log_2_ fold change t_0_) of each transcript for both S and M strain is shown on the y-axis (labeled as S strain) and the x-axis (labeled as M strain), respectively. Blue dots represent the expression of all transcripts at 30m in sporulation. Red dots represent their expression at all the other time-points during sporulation, as indicated. Red line indicates the normal line expected if there was a 100% correlation between the x-axis and y-axis. In 30 min, correlation of expression values between the two strains is high, but the spread keeps on increasing as time progresses.
(PDF)

S6 Fig Expression of sporulation genes in the presence of *MKT1(89G)*. Boxplot showing enrichment of sporulation genes in M strain in comparison to S strain. *P* = 1.96 x 10^–37^ (permutation *P* = 0.16).
(PDF)

S7 Fig Expression profiles for landmark meiotic genes: *IME1*, *NDT80*, *CLB5* and *DIT1*. (A) Sporulation cascade and temporal heat map of meiotic regulators in M and S strains. (B) The expression (log_2_ fold change t_0_) for the meiotic landmark genes is given in the y-axis and the x-axis denotes the time in sporulation medium. Blue line represents the expression of the respective gene in S strain and red line is the same in M strain.
(PDF)

S8 Fig Expression profile for *DAL82*. The expression (log_2_ fold change t_0_) of *DAL82* is given in the y-axis and the x-axis denotes the time in sporulation medium. Blue line represents the expression of *DAL82* in S strain and red line is its expression in M strain.
(PDF)

S9 Fig Heatmaps showing differentially expressed target genes of *DAL82* and *RTG1*. Heatmaps showing expression profiles for the differentially expressed targets genes of *DAL82* and *RTG1* in M and S strain during the course of sporulation (x-axis). These are the same genes as shown Fig 4 and mentioned in S8 Table.
(PDF)

S10 Fig Heatmaps showing all the target genes of *PUF3*, *DAL82* and *RTG1*. Heatmap showing expression profiles for all the known target genes of *PUF3* as given in [44]. Only 13 of 214 genes are differentially expressed, and none of them during early time-points. Heatmaps showing expression profiles for all the target genes of *RTG3* and *DAL82*, in M and S strain. The list of target genes was obtained from YEASTRACT [76]
(PDF)

S1 Table Background SNPs. (XLS)

S2 Table Normalized expression data. (XLS)

S3 Table Smoothed normalised expression data (*locfit*). (XLS)

S4 Table Differentially expressed transcripts (EDGE). (XLS)

S5 Table Genes in each cluster (TimeClust). (XLS)

S6 Table Unique early (Cluster I) genes of the M strain. (XLS)

S7 Table Transcription factors regulating unique early (Cluster I) genes of the M strain. (XLS)

S8 Table Expression values of differentially expressed target genes of *RTG1* and *DAL82* in early time points in the M and S strains. (XLS)

S9 Table Strain names. (XLS)

S10 Table Primer names. (XLS)

S1 Text Supporting methods. (PDF)

S1 File Modeling analysis for progression through meiotic phases. (ZIP)

S2 File Sporulation efficiency data and analysis. (ZIP)

S3 File R scripts for expression data analysis. (ZIP)
