# Supplementary figures and images for: Temporal Expression Profiling Identifies Pathways Mediating Effect of Causal Variant on Phenotype
Source: PLoS Genet. 2015 Jun 3;11(6):e1005195. doi: 10.1371/journal.pgen.1005195 (PMC4454590; doi:10.1371/journal.pgen.1005195)

Figure S1

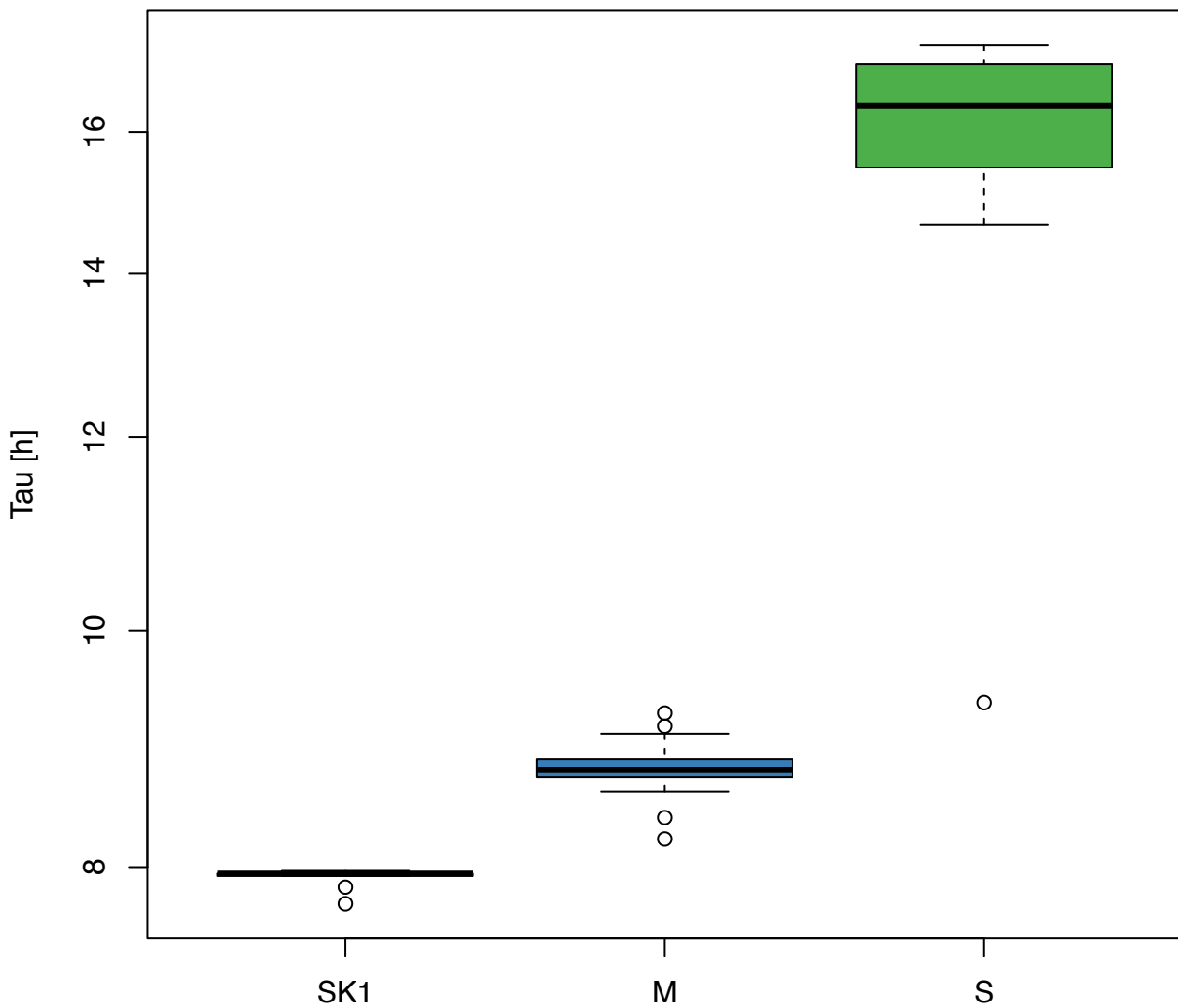

Supplement: S1 Fig — Boxplot showing the initial lag phase (in hours) of the strains (x-axis) in entering meiosis I, calculated by the parameter tau (y-axis). See Methods for details of modeling. (PDF) [file pgen.1005195.s001.pdf]

Figure S2

A

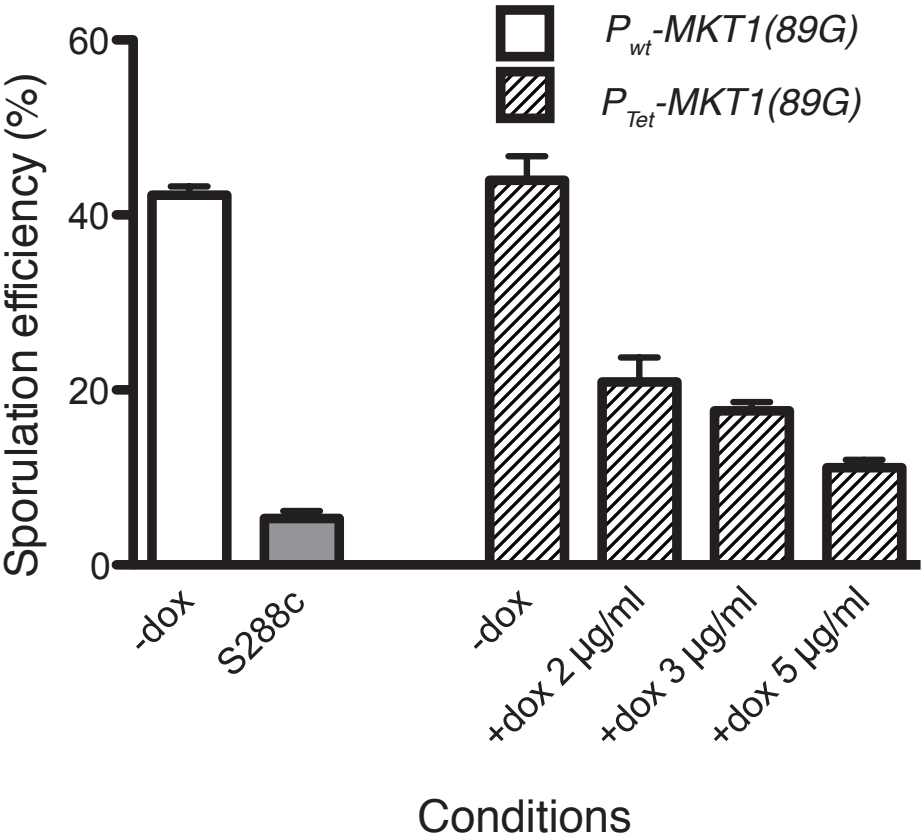

B

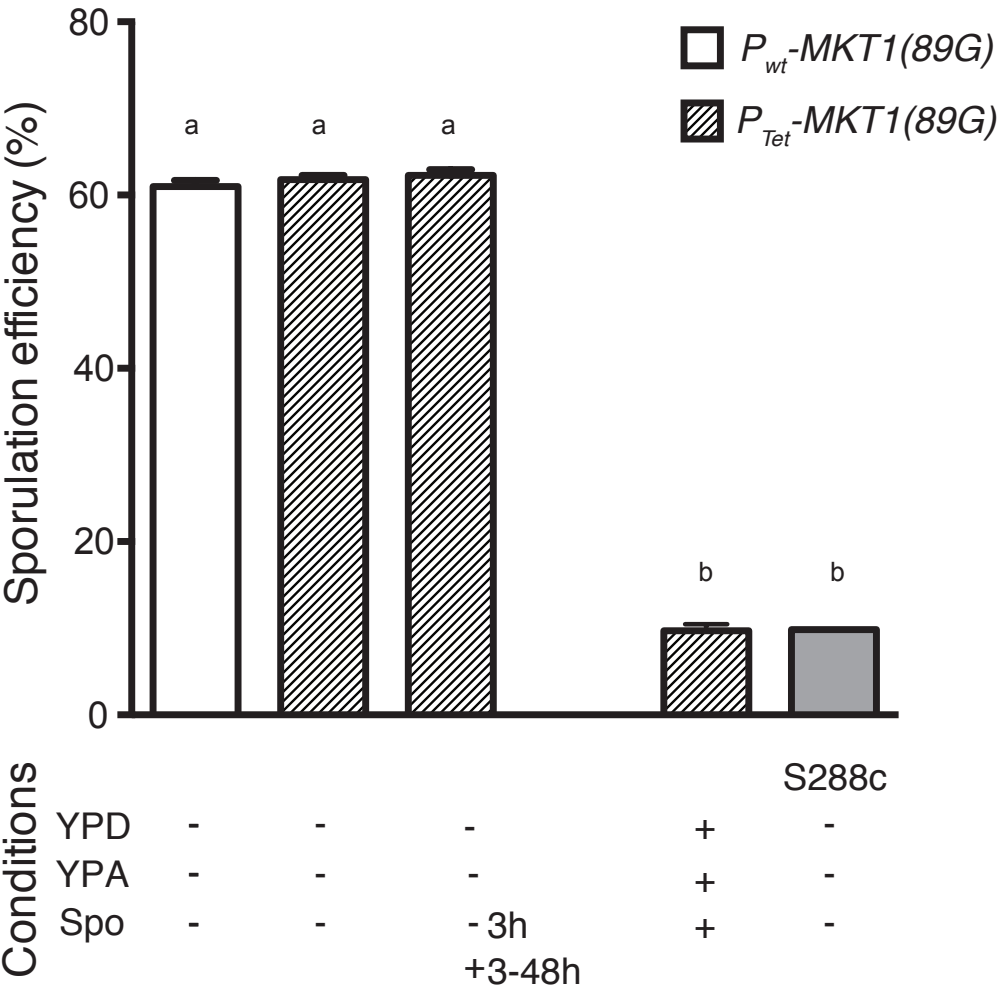

Supplement: S2 Fig — Each strain was grown sequentially in rich (YPD) and pre-sporulation medium (YPA) before incubating in sporulation medium (Spo) for 48h after which sporulation efficiency was estimated. Bar plot represent the mean sporulation efficiency after 48h. (A) Testing doxycycline concentration for switching off MKT1 expression during sporulation. MKT1 expression was switched OFF in all the three conditions by addition of doxycycline (indicated as +dox). No doxycycline in any of the three media is indicated as-dox (implying MKT1 expression ON). Concentration of doxycycline is depicted on x-axis. Tukey’s multiple comparisons test (P < 0.05) was performed. In both concentrations 3μg/ml and 5μg/ml of doxycycline, M strain showed sporulation efficiency equivalent to S strain. 2μg/ml doxycycline showed significant difference in mean sporulation efficiency compared to S strain. Further experiments were performed using 5μg/ml doxycycline. Error bars are the standard errors of mean. (B) Early role of MKT1 expression. MKT1 expression was switched OFF by addition of doxycycline (indicated as +), and MKT1 expression was ON when no doxycycline was added (indicated as-). “-3h” condition indicates that no doxycycline was added till 3h in sporulation medium. “+3-48h” condition indicates that MKT1 expression was switched OFF 3h-post initiation of sporulation, by adding doxycycline during 3–48h in sporulation medium. Tukey’s multiple comparisons test (P < 0.05), bars with the same letter code do not differ significantly. Error bars are the standard errors of mean. (PDF) [file pgen.1005195.s002.pdf]

Figure S3

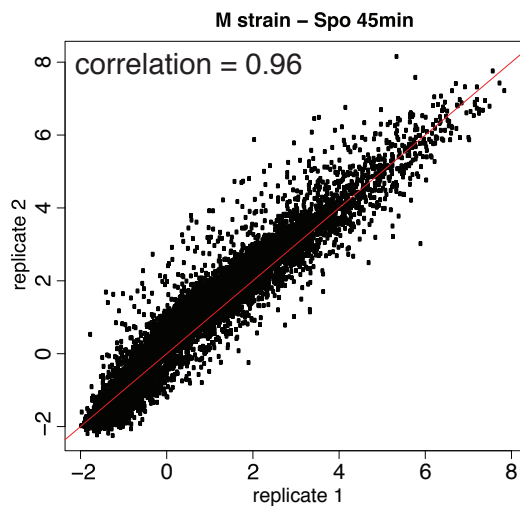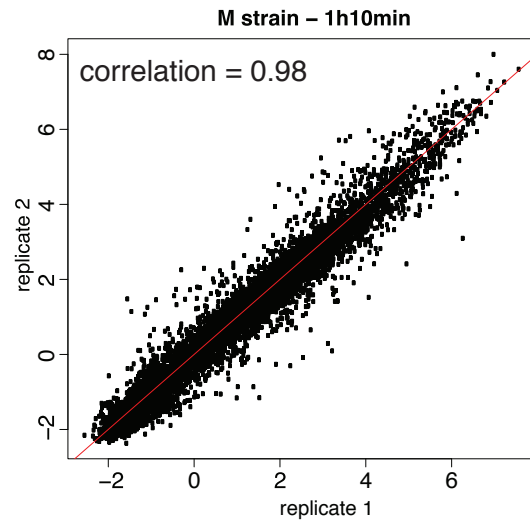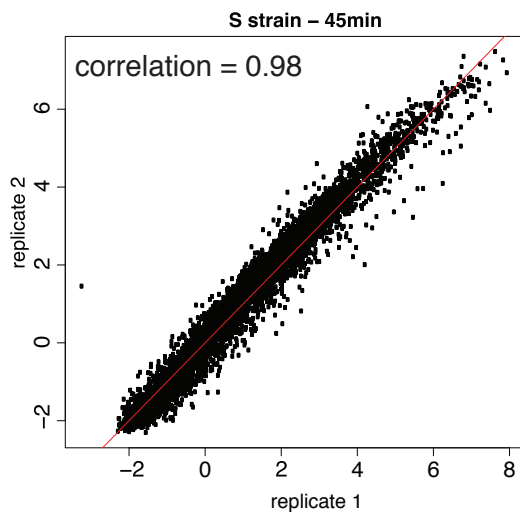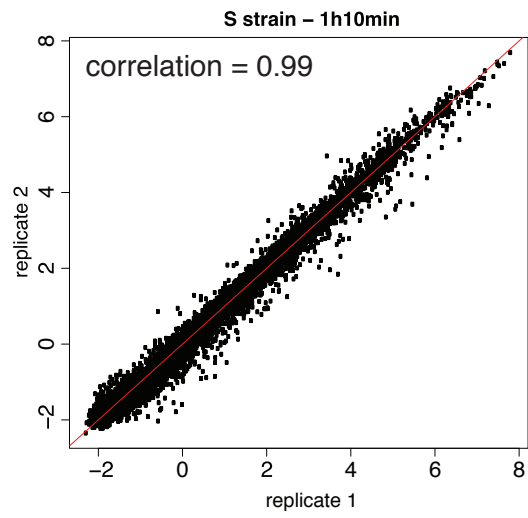

Supplement: S3 Fig — The expression for each transcript in the two replicates has been plotted against each other. Replicate 1 is in x-axis and replicate 2 is in y-axis. Red line indicates the normal line expected if there was a 100% correlation between the replicates. (PDF) [file pgen.1005195.s003.pdf]

Figure S4

M strain

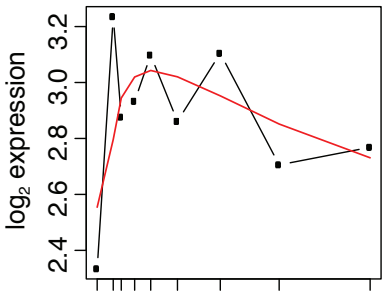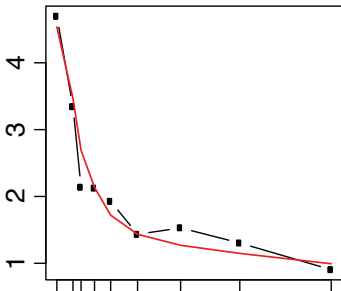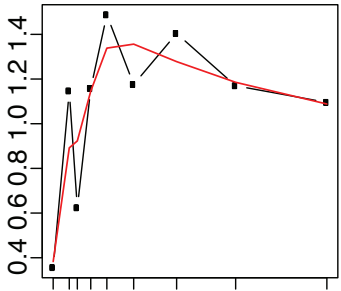

S strain

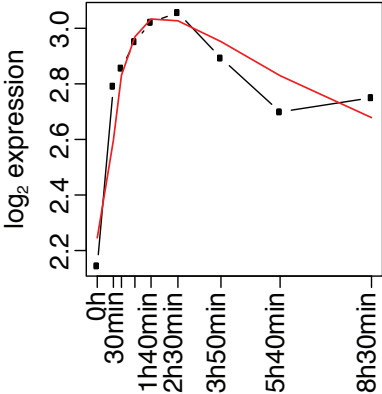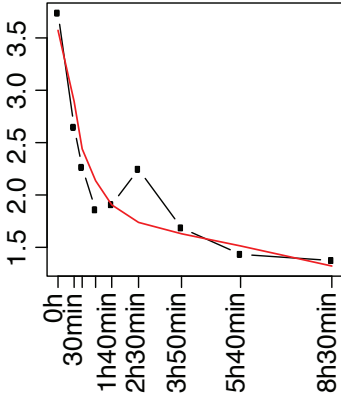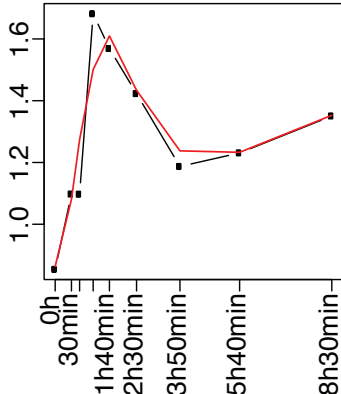

*RCR2*

*URA1*

*LST7*

Supplement: S4 Fig — Representative images showing normalized (black line) and normalized locfit (red line) data in M and S strain. x-axis denotes the time-points in sporulation medium and y-axis is the log2 expression. (PDF) [file pgen.1005195.s004.pdf]

Figure S5

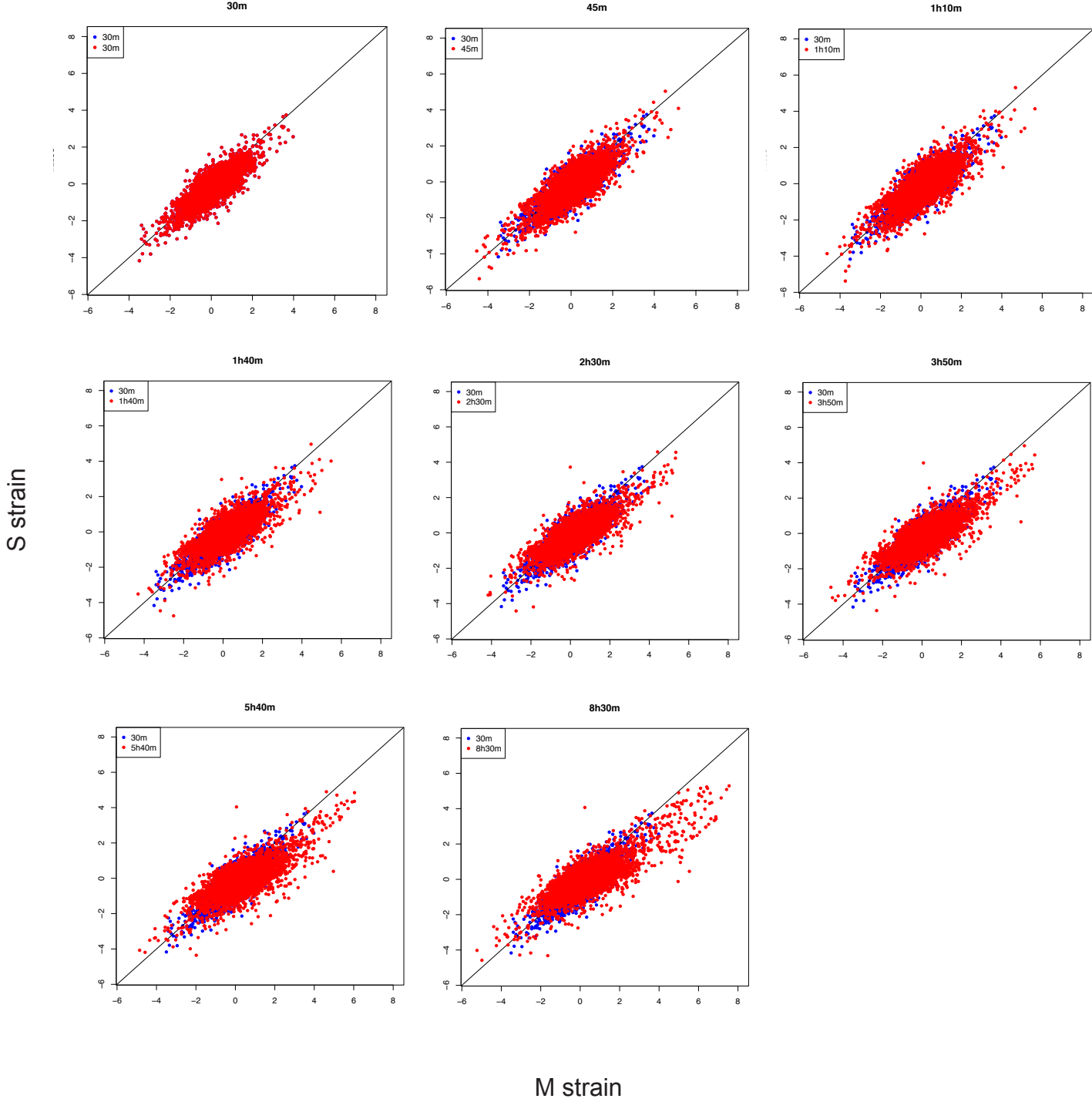

Supplement: S5 Fig — The expression (log2 fold change t0) of each transcript for both S and M strain is shown on the y-axis (labeled as S strain) and the x-axis (labeled as M strain), respectively. Blue dots represent the expression of all transcripts at 30m in sporulation. Red dots represent their expression at all the other time-points during sporulation, as indicated. Red line indicates the normal line expected if there was a 100% correlation between the x-axis and y-axis. In 30 min, correlation of expression values between the two strains is high, but the spread keeps on increasing as time progresses. (PDF) [file pgen.1005195.s005.pdf]

Figure S6

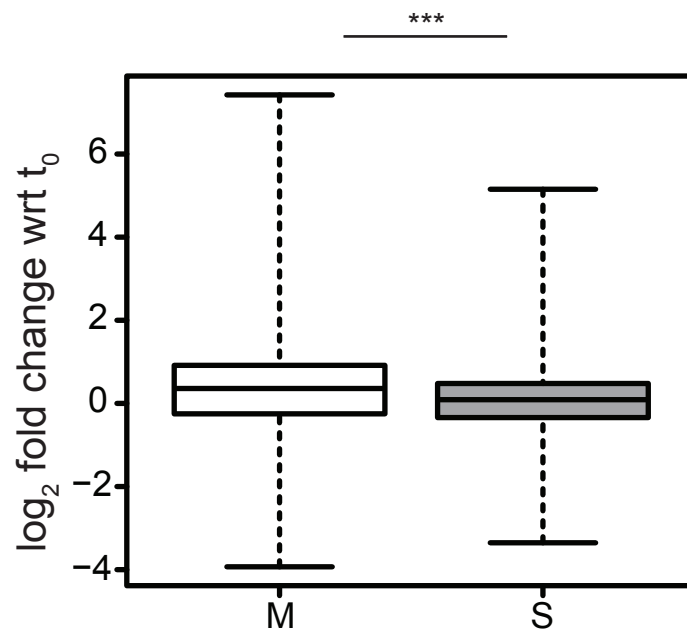

Supplement: S6 Fig — Boxplot showing enrichment of sporulation genes in M strain in comparison to S strain. P = 1.96 x 10^–37^ (permutation P = 0.16). (PDF) [file pgen.1005195.s006.pdf]

Figure S7

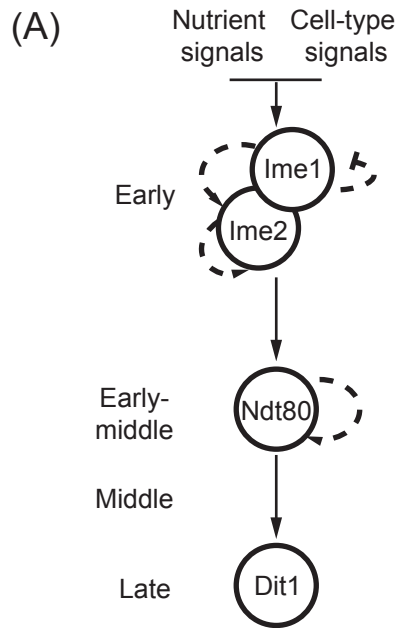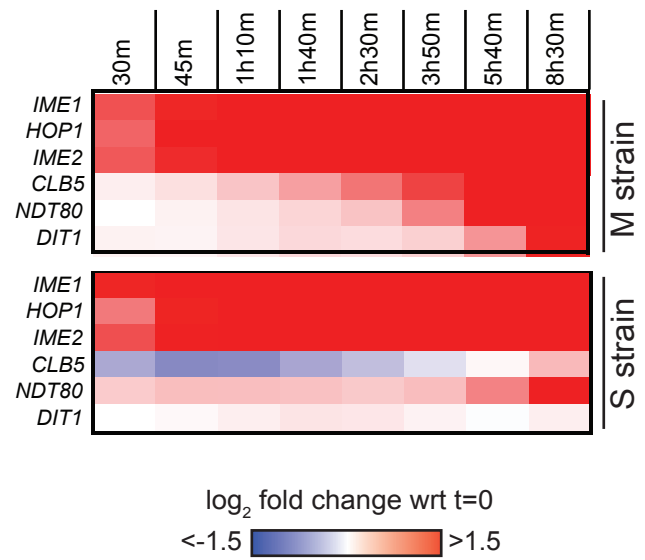

(B)

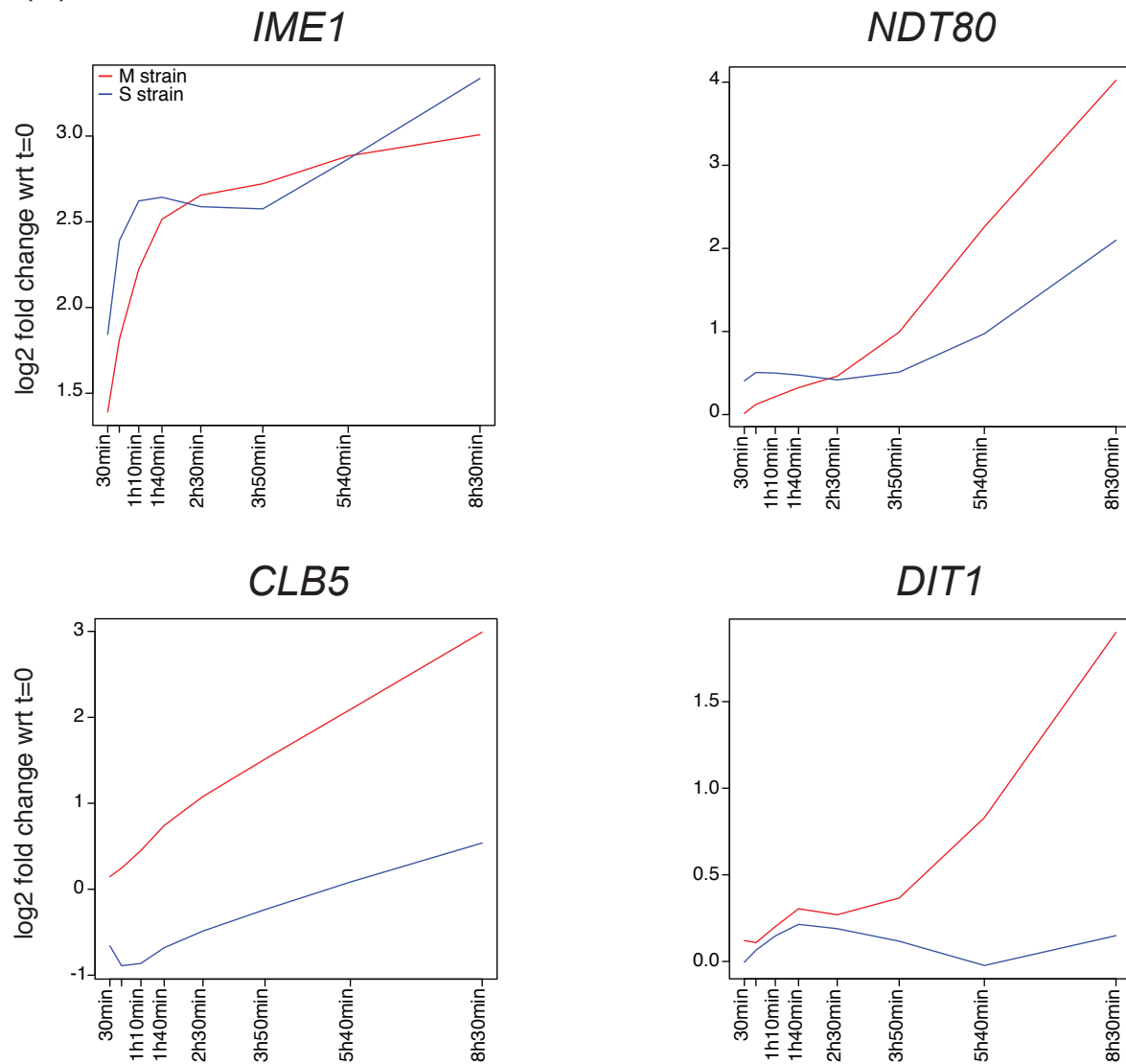

Supplement: S7 Fig — (A) Sporulation cascade and temporal heat map of meiotic regulators in M and S strains. (B) The expression (log2 fold change t0) for the meiotic landmark genes is given in the y-axis and the x-axis denotes the time in sporulation medium. Blue line represents the expression of the respective gene in S strain and red line is the same in M strain. (PDF) [file pgen.1005195.s007.pdf]

Figure S8

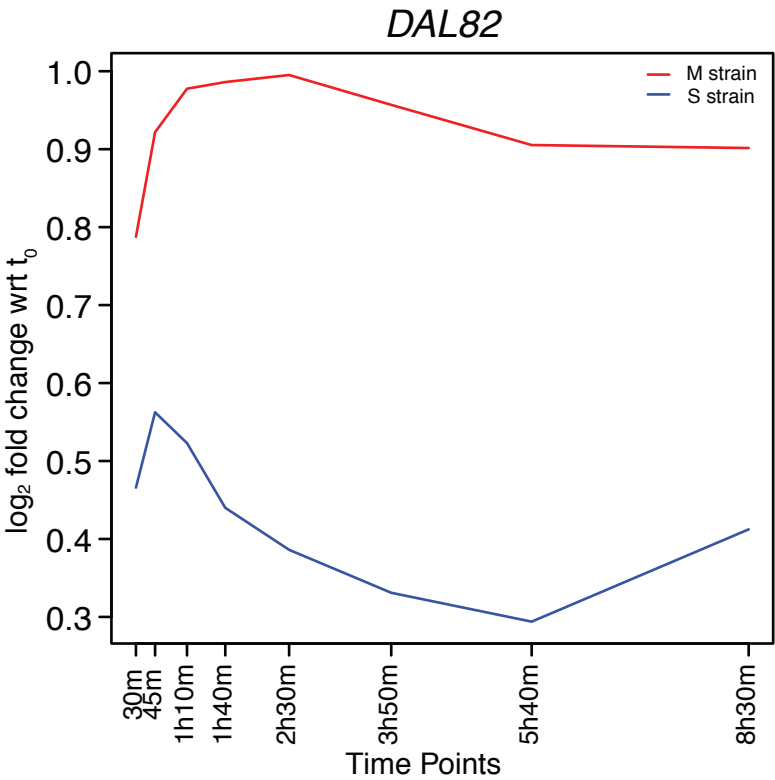

Supplement: S8 Fig — The expression (log2 fold change t0) of DAL82 is given in the y-axis and the x-axis denotes the time in sporulation medium. Blue line represents the expression of DAL82 in S strain and red line is its expression in M strain. (PDF) [file pgen.1005195.s008.pdf]

Figure S9

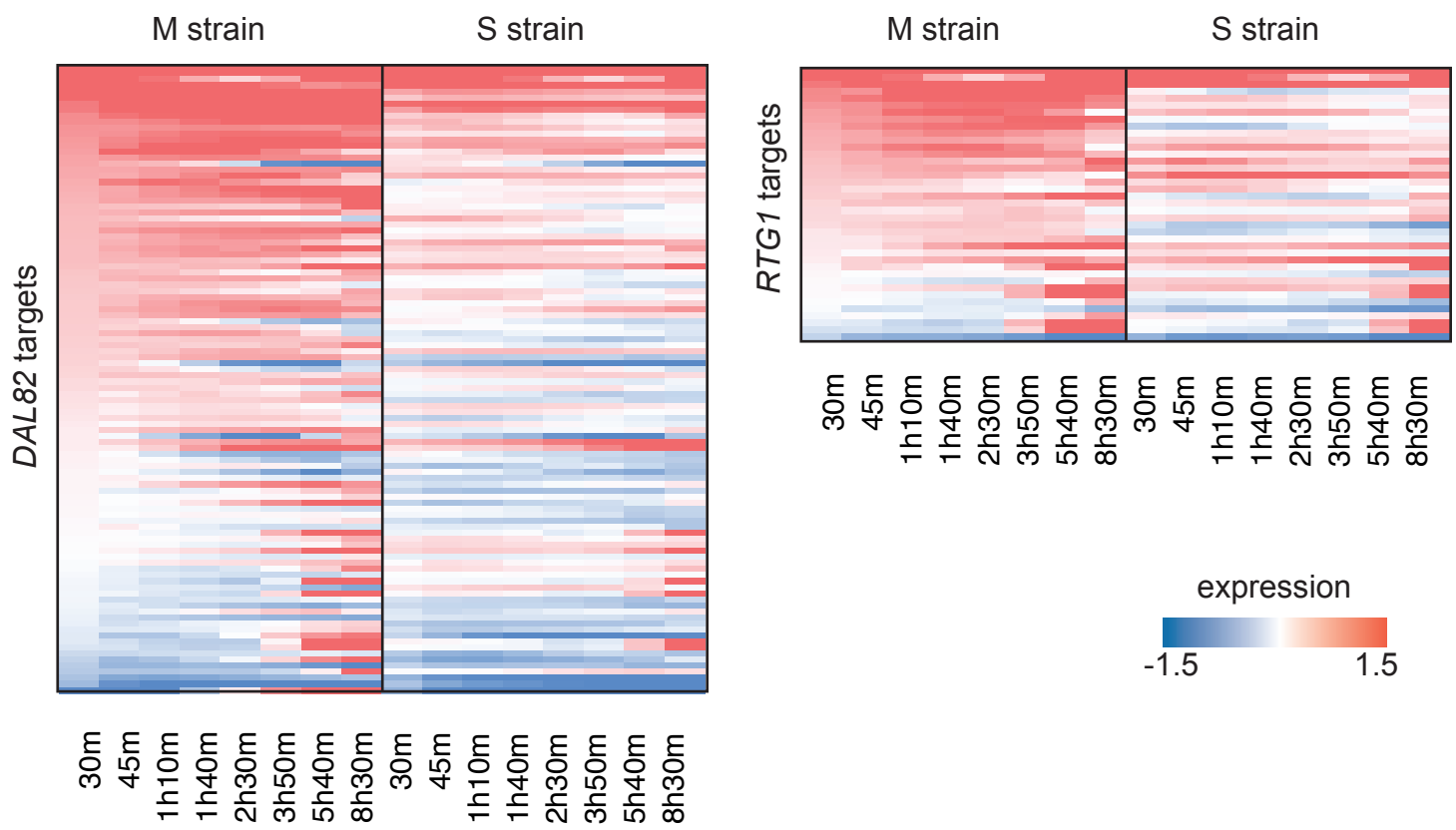

Supplement: S9 Fig — Heatmaps showing expression profiles for the differentially expressed targets genes of DAL82 and RTG1 in M and S strain during the course of sporulation (x-axis). These are the same genes as shown Fig 4 and mentioned in S8 Table. (PDF) [file pgen.1005195.s009.pdf]

Figure S10

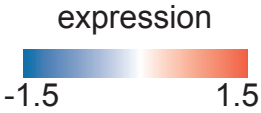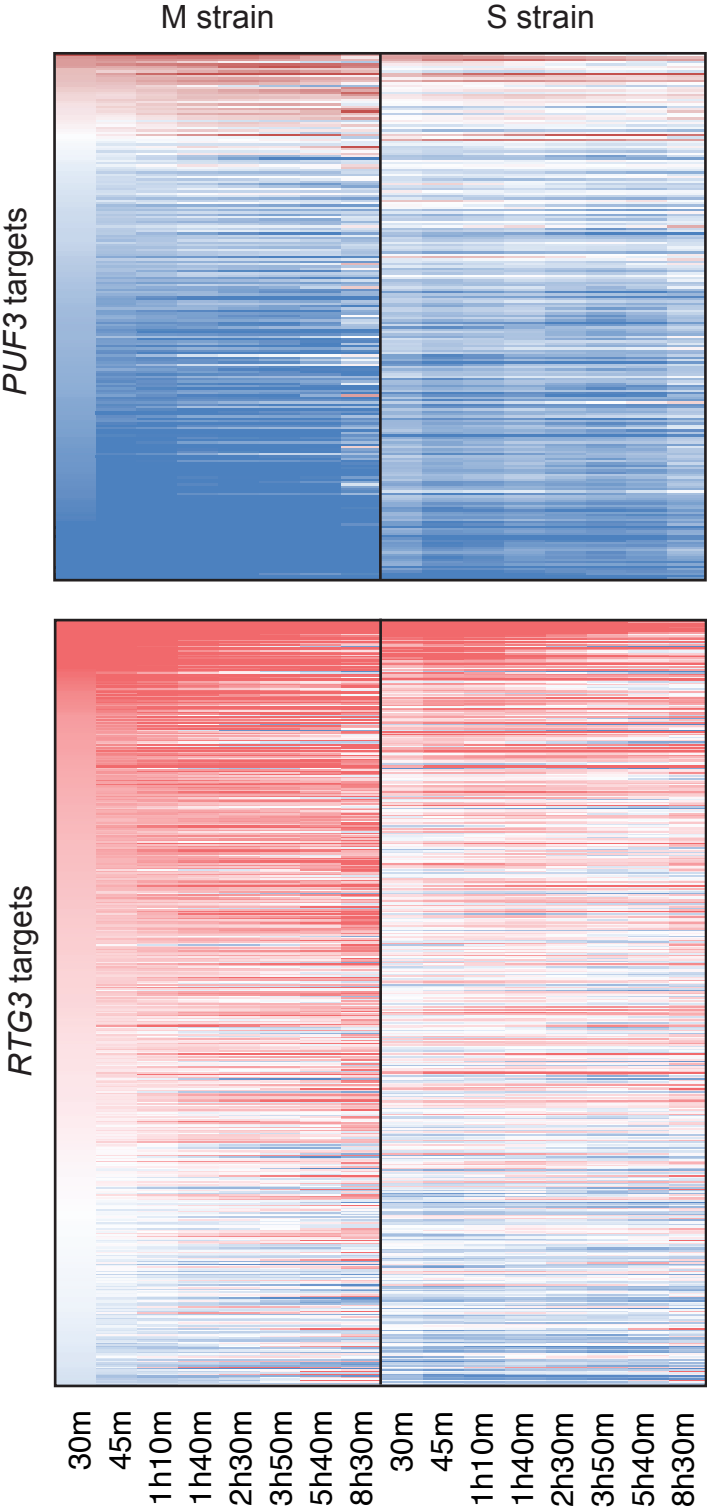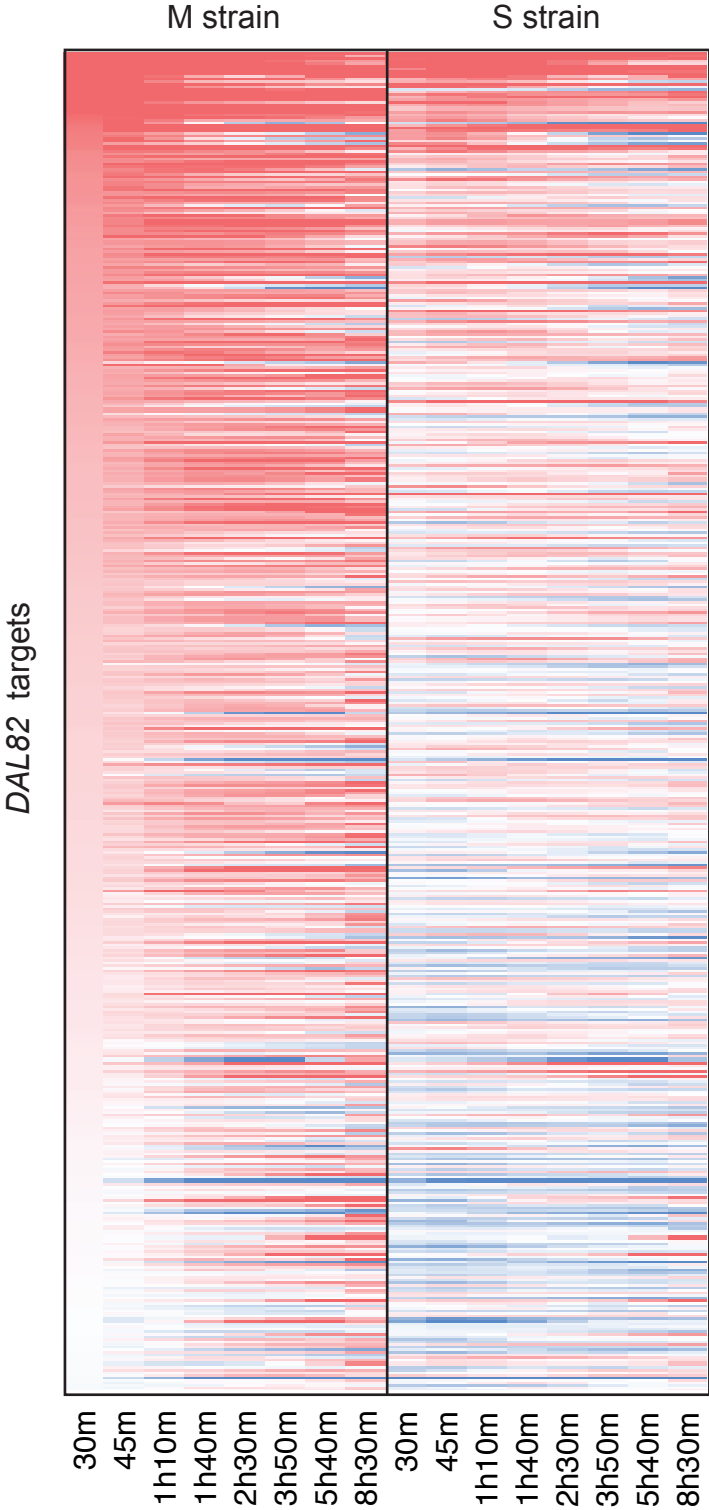

Supplement: S10 Fig — Heatmap showing expression profiles for all the known target genes of PUF3 as given in [44]. Only 13 of 214 genes are differentially expressed, and none of them during early time-points. Heatmaps showing expression profiles for all the target genes of RTG3 and DAL82, in M and S strain. The list of target genes was obtained from YEASTRACT [76] (PDF) [file pgen.1005195.s010.pdf]
