# Supplementary material for: Temporal Expression Profiling Identifies Pathways Mediating Effect of Causal Variant on Phenotype
Source: PLoS Genet. 2015 Jun 3;11(6):e1005195. doi: 10.1371/journal.pgen.1005195 (PMC4454590; doi:10.1371/journal.pgen.1005195)
Supplement: S1 Text — (PDF) [file pgen.1005195.s021.pdf]

Temporal Expression Profiling Identifies Pathways Mediating Effect of Causal Variant on Phenotype

Gupta S, Radhakrishnan A, Pandu R-L, Gen L, Steinmetz LM, Gagneur J and Sinha H

S1 Text

Supplementary Methods

Section 1: Whole-genome sequencing of allele replacement strain relative to S288c strain

Presence of the causative single nucleotide polymorphism (SNP), *MKT1(89G)*, in the allele replacement strain YAD351 [15] was confirmed by whole-genome re-sequencing with S288c as the reference. The yeast strains were grown in YPD medium and genomic DNA was isolated using PrepEase Genomic DNA Isolation Kit (Affymetrix) according to the manufacturer's protocol. 10 micrograms of genomic DNA was fragmented to a size range of 250-400 bp using a Covaris E series sonicator (Duty cycle - 10%, Intensity - 4.5, Cycles per burst - 200, 60 sec). End repair, dA tailing, ligation and amplification were done according to Illumina library prep protocol and the libraries were size selected for 350 bp using an invitrogen E-gel sizeselect 2% agarose gel. The libraries were sequenced using Illumina Hiseq 2000 at the European Molecular Biology Laboratory Genomics core facility (Genecore).

For calling SNPs and mutations in the samples, reads were aligned to the S288c reference genome (build R63) using Novoalign (v2.07.06; http://www.novocraft.com/ *webcite*), allowing for unique alignments. The parental S96 strain was also sequenced and its reads were processed similarly. Subsequent SNP calling was performed on all samples using SAMtools [78]. The vcf file produced by SAMtools contains a list of individual genotype calls across all samples at each variant position. To identify mutations in the derived strains, genotype calls were compared at variants with quality >= 30. A total of 12 variant positions (including the causative *MKT1* allele on Chr IV) were found where the genotype call in the parental strain differs from that in the parental S288c strain, of which 5 were homozygous (S1 Table).

Section 2: Backcross strategy to remove secondary SNPs from allele replacement strain

Since the secondary SNPs identified from whole-genome re-sequencing could confound our analyses, three consecutive backcrosses with the reference haploid S288c strain were done to remove these SNPs. YAD351(MATa) was backcrossed to S288c(MATα) parent strain (backcross 1, BC1). From this cross, we identified a single segregant with *MKT1(89G)*(MATa) SNP by sequencing 650 bp PCR-fragment spanning the SNP. This segregant was then backcrossed to the S288c(MATα) parent strain (backcross 2, BC2). Following three consecutive backcrosses, the

haploid BC3 (backcross 3) segregants obtained after tetrad dissection were tested for the five secondary mutations identified from whole-genome sequencing. A 650 bp region around these secondary mutations was sequenced to identify a clean S288c strain with only *MKT1(89G)*. This strain was diplodized using pHS2 plasmid (containing a functional *HO*) and is called as the M strain in our study.

Section 3: Normalization of the time-series expression data

M strain, S288c, a non-sporulating S288c (MATa/Δ) strain, allele replacement strain with *TAO3* causative SNP were normalised using *vsn* normalization method [73]. However, since for this study we have used only the M strain and S288c arrays, further analyses was performed using their normalized expression data (S2 Table). A few of the arrays (for time points 45min and 1h10min of M and S strain) were replicated. Correlations between the normalized expression values of replicates were greater than 0.95 for both the strains, confirming reproducibility between the replicates (S3 Fig.). Hence, further analysis was performed using single replicate data of M and S strains.

Section 4: Smoothing of normalised temporal data using *locfit*

The top 10% of the transcripts ordered by the descending order of variability in expression values were considered for the optimization of '*h*'. Optimized '*h*' was estimated as the value that gave minimum error by the leave one out method for a range of bandwidths. In a big range between 1 and 20, the bandwidth with minimum error was mainly observed to be between 1 and 3. Thus, this was used as the next range. In the range between 1 and 3, the two most optimum bandwidth were observed to be h=1.21 & h=3. A random set of 20 transcripts were selected and plotted to select the optimum '*h*' (few shown in S4 Fig.). h=1.21 was selected since the transcripts were observed to be over-smoothened when h=3 as when compared to h=1.21. locfit data can be seen in S3 Table.

References

78. Li H, Handsaker B, Wysoker A, Fennell T, Ruan J, Homer N, et al. The Sequence Alignment/Map format and SAMtools. Bioinformatics. 2002; 25: 2078–2079.
