# Supplementary material for: Temporal Expression Profiling Identifies Pathways Mediating Effect of Causal Variant on Phenotype
Source: PLoS Genet. 2015 Jun 3;11(6):e1005195. doi: 10.1371/journal.pgen.1005195 (PMC4454590; doi:10.1371/journal.pgen.1005195)

S1 File

Gupta S, Radhakrishnan A, Pandu R-L, Gen L, Steinmetz LM, Gagneur J and Sinha H

April 1, 2015

1 Mathematical modeling for progression through meiotic phases

Frequency of cells in with 1, 2 or 4 nuclei (see Fig. A-C below) indicate that the dynamics differ between the backgrounds already at the time cells start to divide (beginning of the drop of the 1-nucleus population) and the rate of this. We considered a model of the sporulation to assess whether these differences are significant, and whether the remaining changes in cell stage frequencies can be simply explain as consequences of the differences in the early sporulation processes or due to differences in the kinetic of later events as well.

1.1 Parametrization of cell stage kinetics

Cells were assumed to be either in a 1-nucleus active state at frequency $X_1(t)$, in a 1-nucleus inactive state at frequency $Y_1(t)$, in a 2-nuclei active state at frequency $X_2(t)$, in a 2-nuclei inactive state at frequency $Y_2(t)$, or in a 4-nuclei state at frequency $X_4(t)$. The existence of inactive states is supported by the fact that at steady state, some cells still have one nucleus or 2 nuclei indicating they are trapped at these stages. We assumed that no other state was possibe, thus these frequencies must sum to 1. The dynamics was modeled with an initial lag phase of duration τ followed by first order kinetics as follows:

$$\text{for all } t \leq \tau : \begin{cases} X_1(t) = 1 \\ X_2(t) = X_4(t) = Y_1(t) = Y_2(t) = 0 \end{cases}$$

, and:

$$\text{for all } t > \tau : \begin{cases} \frac{dX_1}{dt} = -(\alpha + \beta) X_1 \\ \frac{dX_2}{dt} = \alpha X_1 - (\gamma + \delta) X_2 \\ \frac{dX_4}{dt} = \gamma X_2 \\ \frac{dY_1}{dt} = \beta X_1 \\ \frac{dY_2}{dt} = \delta X_2 \end{cases}$$

This model leads to the following closed form solutions:

$$\text{for all } t > \tau : \begin{cases} X_1(t) = e^{-(\alpha+\beta)t'} \\ X_2(t) = \frac{\alpha}{\mu-\lambda}(e^{-t'\lambda} - e^{-t'\mu}) \\ X_4(t) = \frac{\alpha\gamma}{\mu-\lambda}[\frac{1}{\lambda}(1 - e^{-t'\lambda}) - \frac{1}{\mu}(1 - e^{-t'\mu})] \\ Y_1(t) = \frac{\beta}{\alpha+\beta}(1 - e^{-t'(\alpha+\beta)}) \\ Y_2(t) = \frac{\alpha\delta}{\mu\lambda(\mu-\lambda)}(\lambda e^{-t'\mu} - \mu e^{-t'\lambda} + (\mu - \lambda)) \end{cases}$$

, where $t' = t - \tau$, $\lambda = \alpha + \beta$, and $\mu = \gamma + \delta$.

1.2 Model fitting

For each background, the data consisted of frequencies $f_{i,j}$ of cells with $j \in \{1, 2, 4\}$ nuclei measured at time t_i. The model described above was fitted to minimize the sum of squared errors:

$$\min_{\boldsymbol{\theta}} \sum_i (f_{i,1} - X_1(t_i) - Y_1(t_i))^2 + (f_{i,2} - X_2(t_i) - Y_2(t_i))^2 + (f_{i,4} - X_4(t_i))^2$$

where $\boldsymbol{\theta} = (\alpha, \beta, \gamma, \delta, \tau)^T$ is the vector of parameters. The cost function was minimized using the R function `optim()` with default parameters. We parametrized the cost function with the logarithm of the parameters to ensure their positivity (these are lag times and rates). Fits are shown in Fig. A-C below.

1.3 Confidence intervals

Confidence intervals on the parameters were obtained with a leave-one-out approach, where the replicate data for each time point one at a time were left out and the model fitted on the remaining time points (see Fig. D-H below). We found that this bootstrapping scheme was more stringent and more appropriate than sampling with replacement the complete dataset without stratifying per time point because frequency measurements within one time point were closer to each other than to the fitted values.

1.4 Conclusion

The results show that the only significant difference was observed for τ parameter (see Fig. H below).

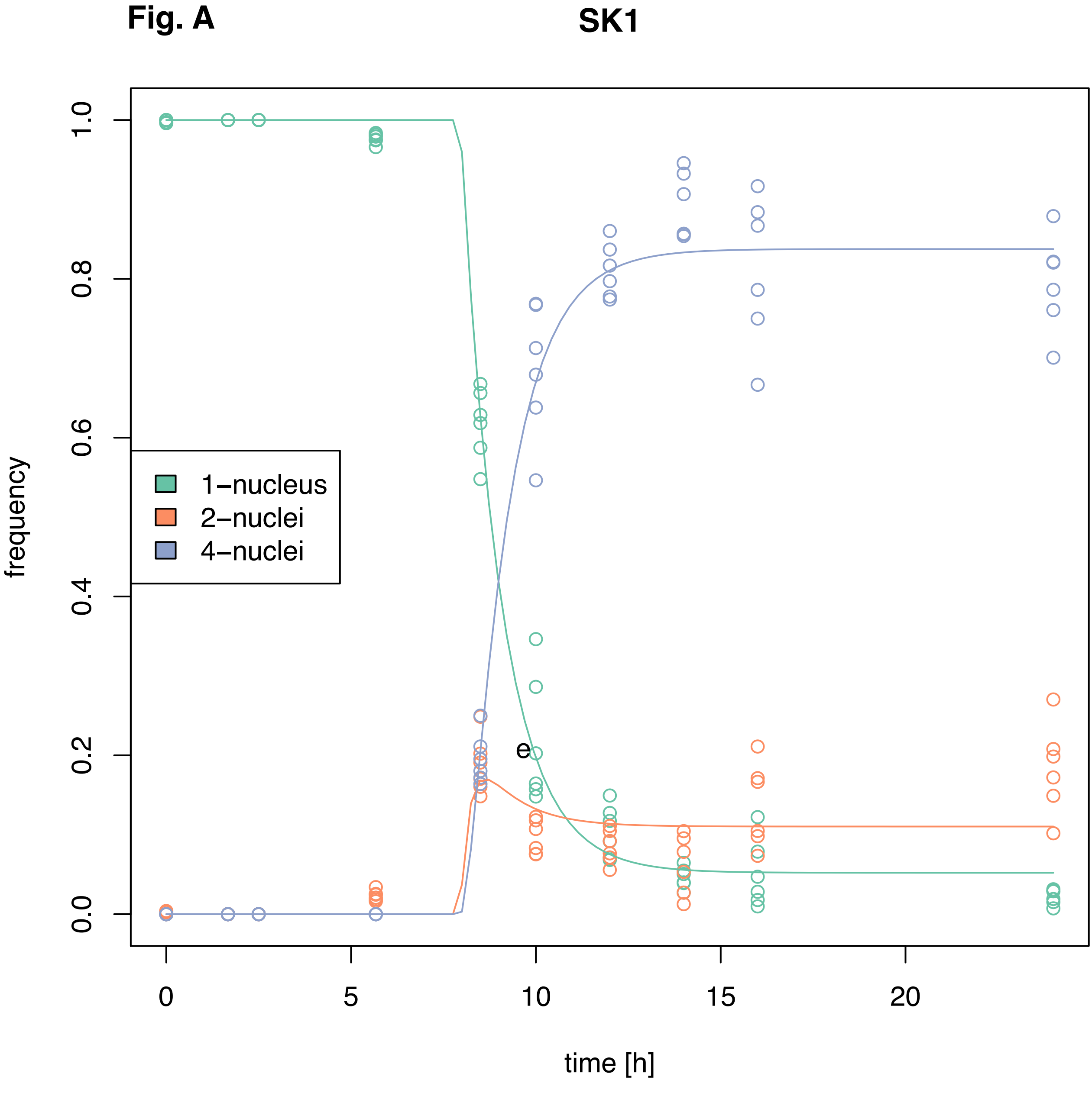
Fig. A
SK1
1−nucleus
2−nuclei
4−nuclei
frequency
time [h]
e
0.0
0.2
0.4
0.6
0.8
1.0
0
5
10
15
20

Fig. B

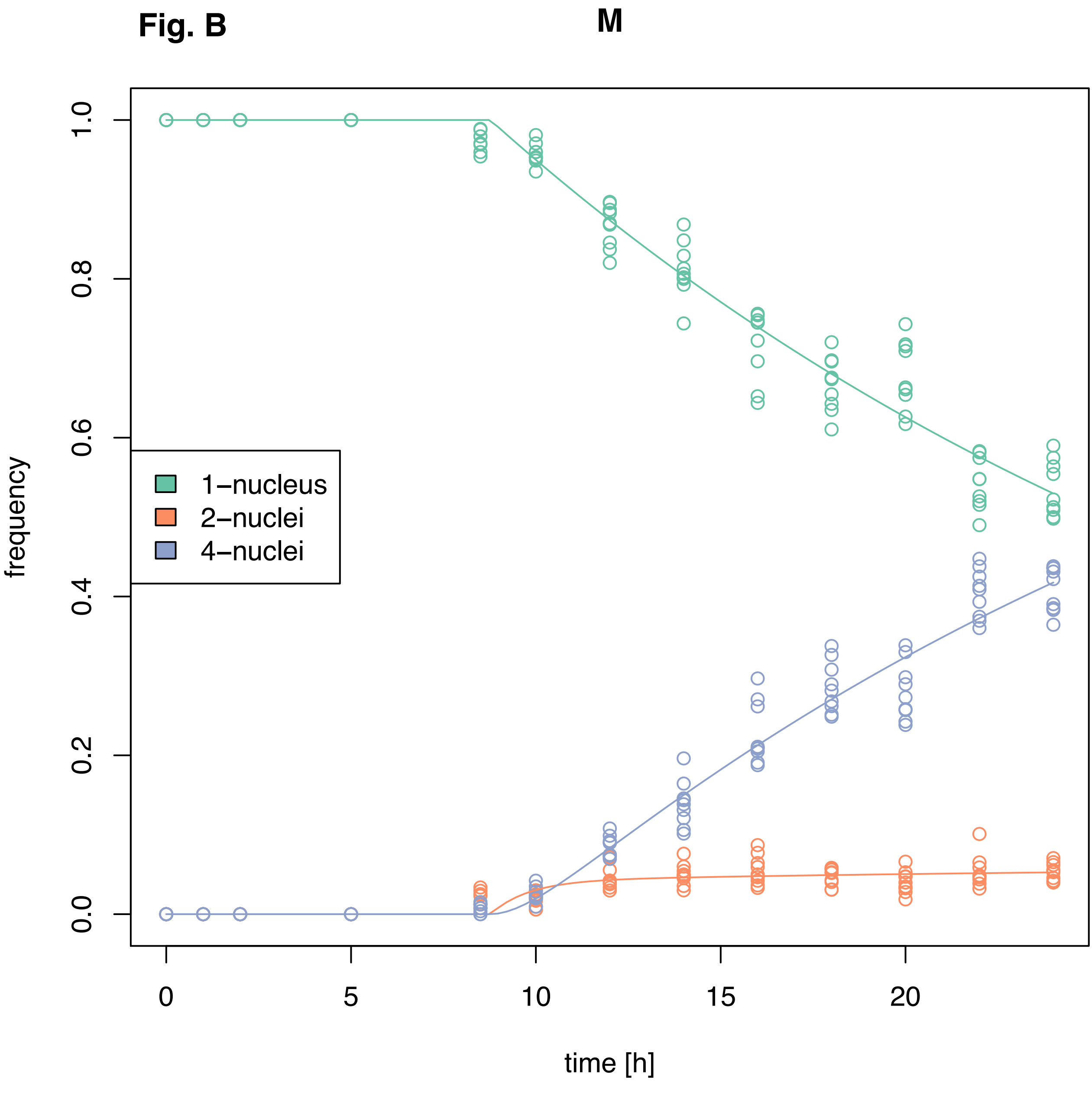

M
1−nucleus
2−nuclei
4−nuclei
frequency
0.0
0.2
0.4
0.6
0.8
1.0
time [h]
0
5
10
15
20

Fig. C

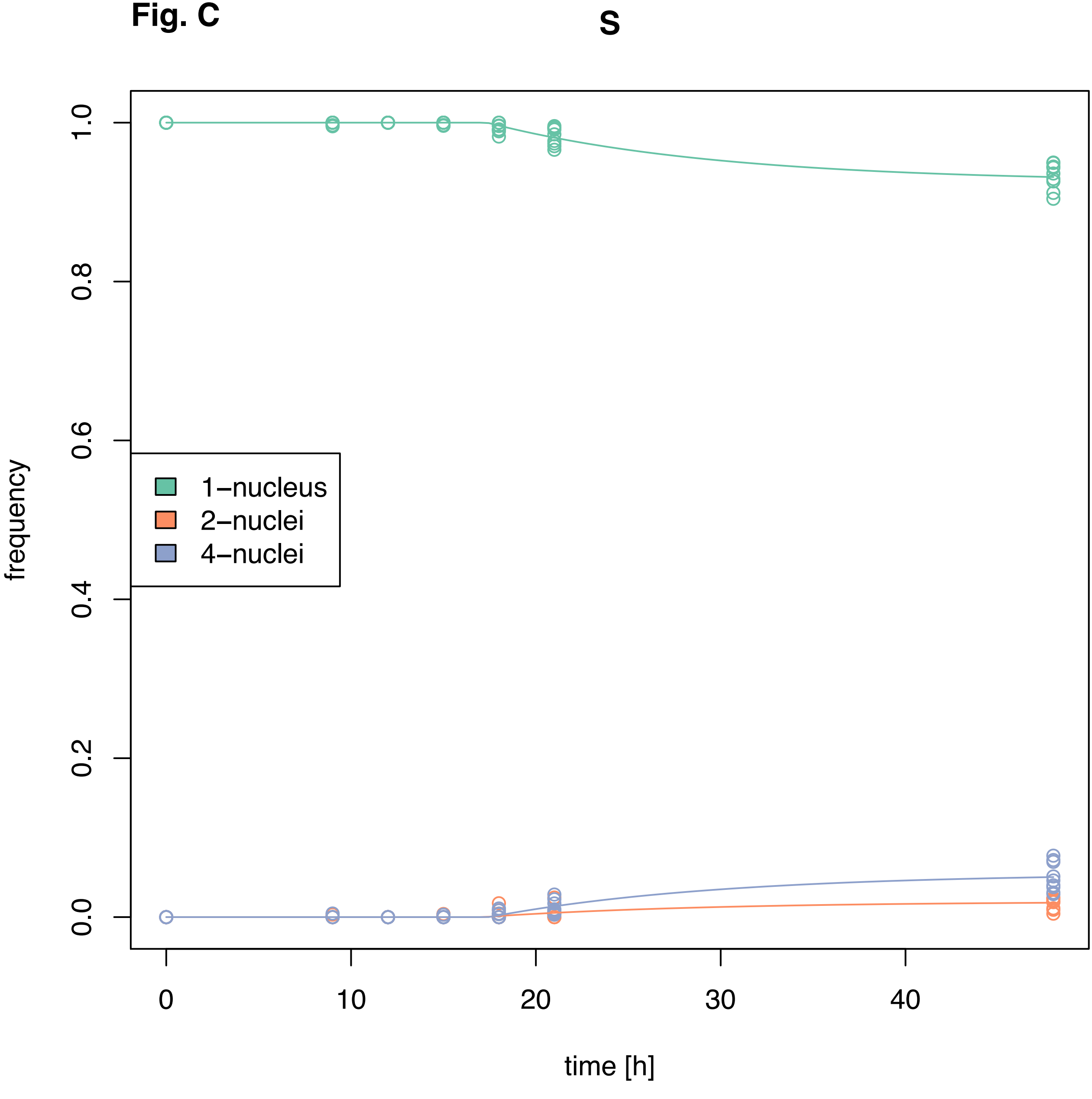
S
1−nucleus
2−nuclei
4−nuclei
frequency
0.0
0.2
0.4
0.6
0.8
1.0
0
10
20
30
40
time [h]

Fig. D

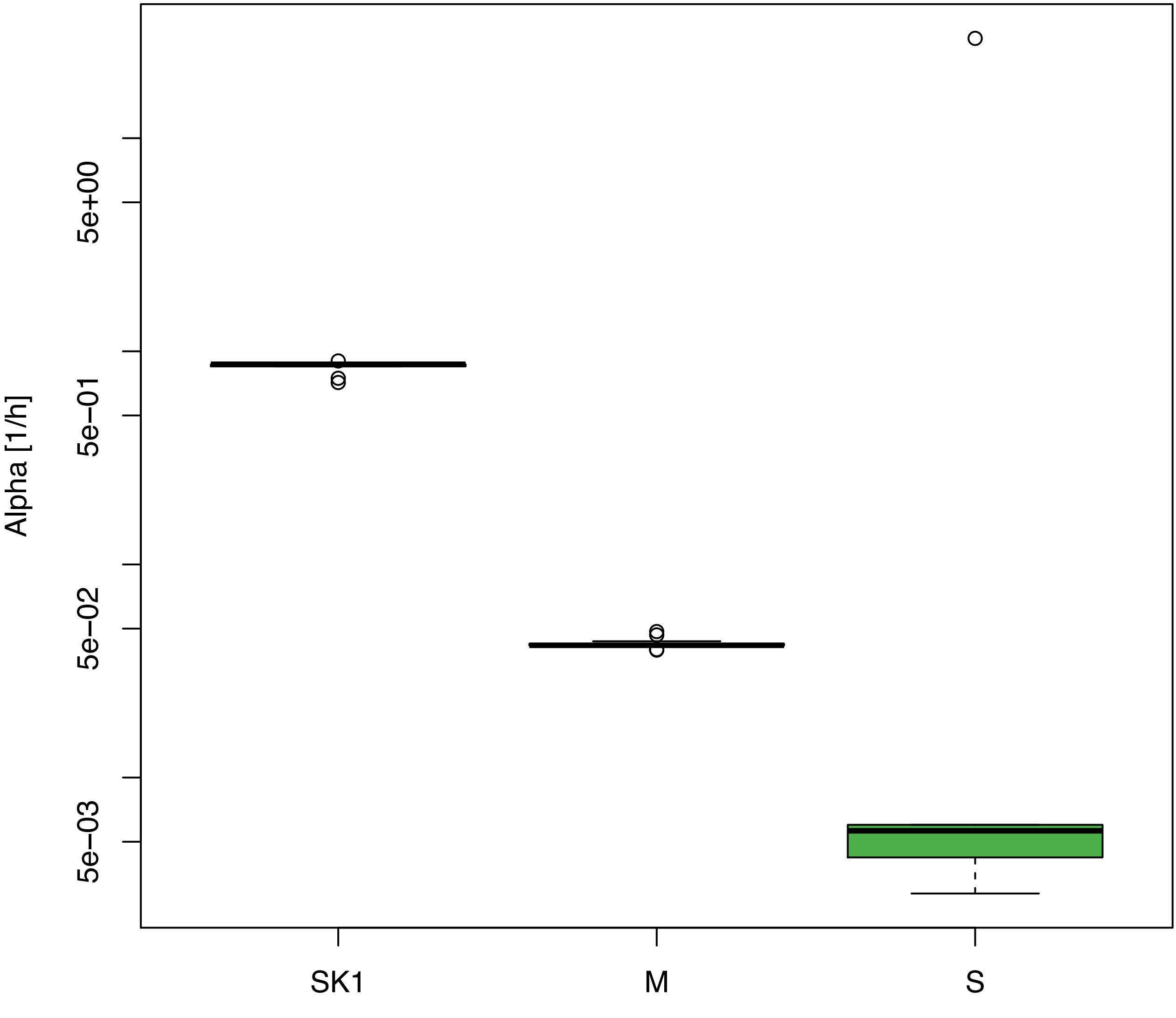

Fig. E

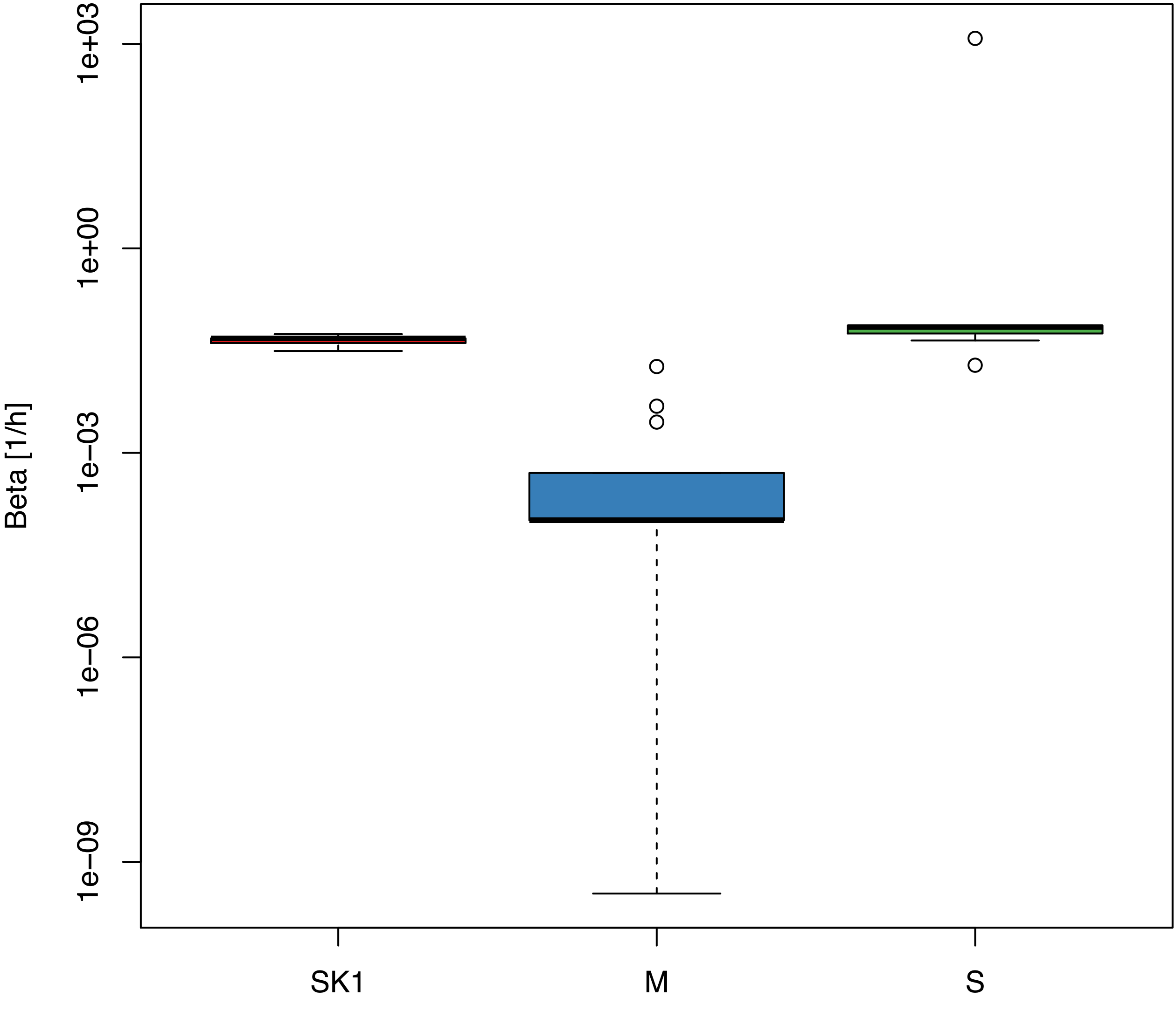

Fig. F

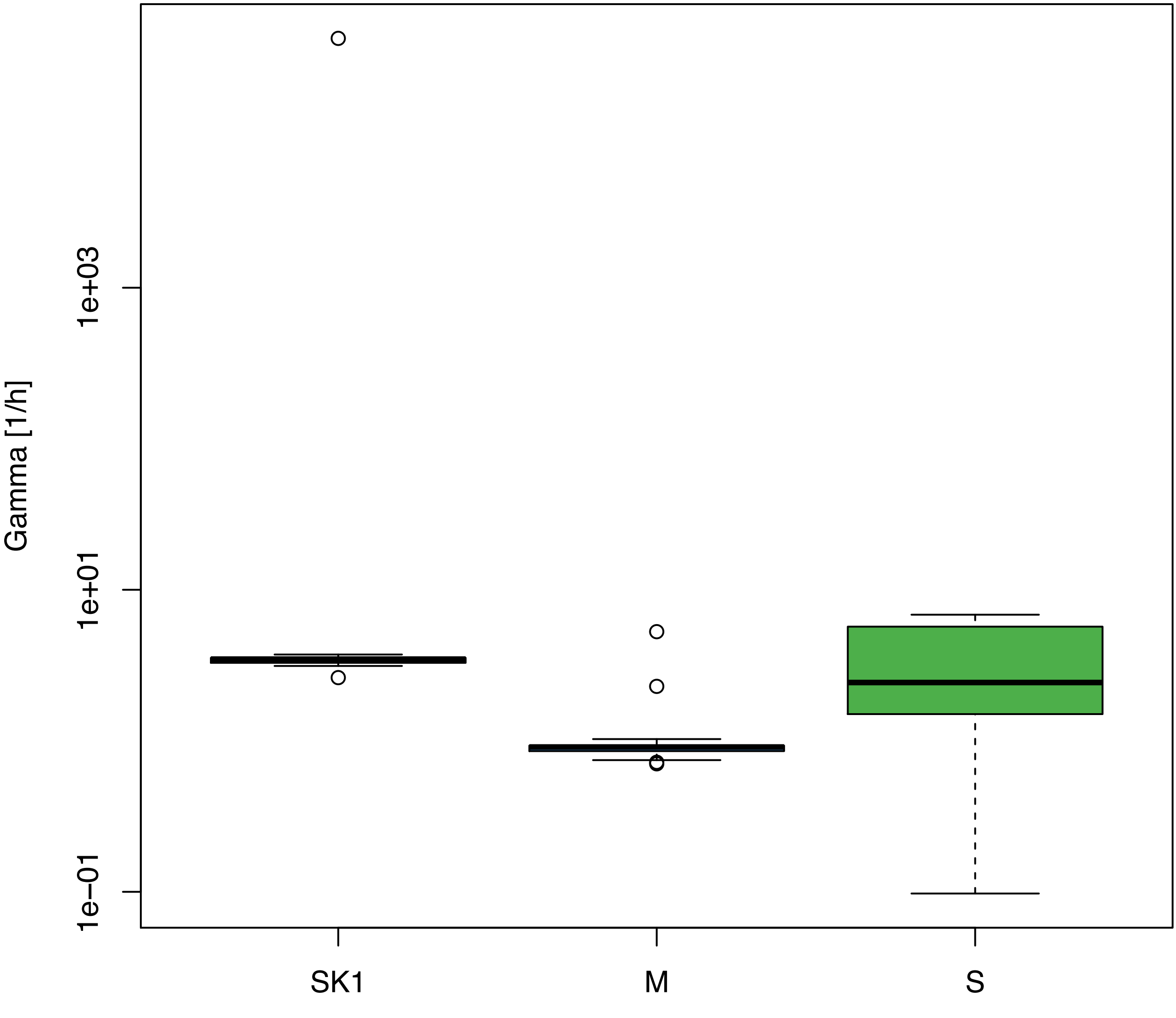

Fig. G

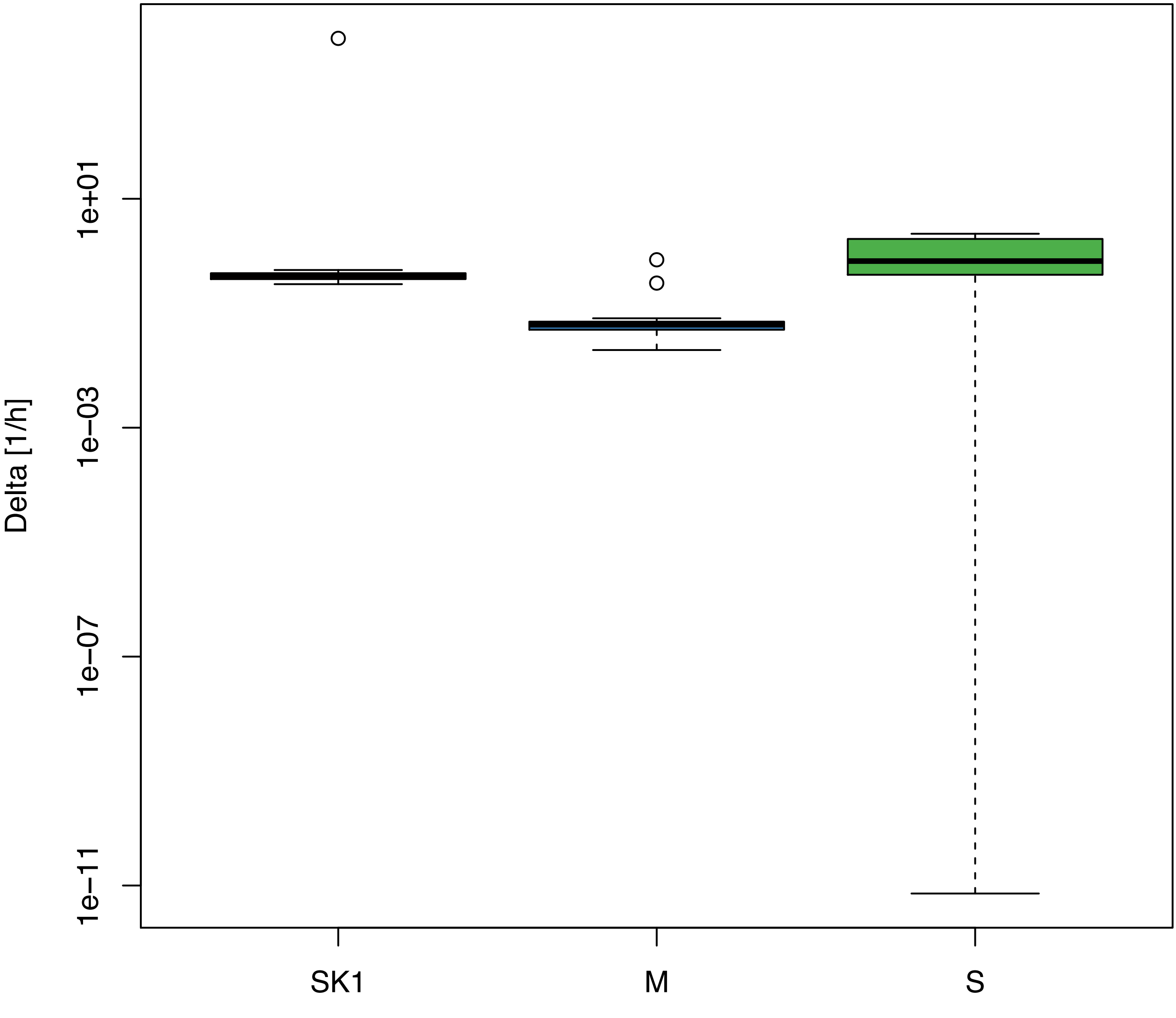

Fig. H

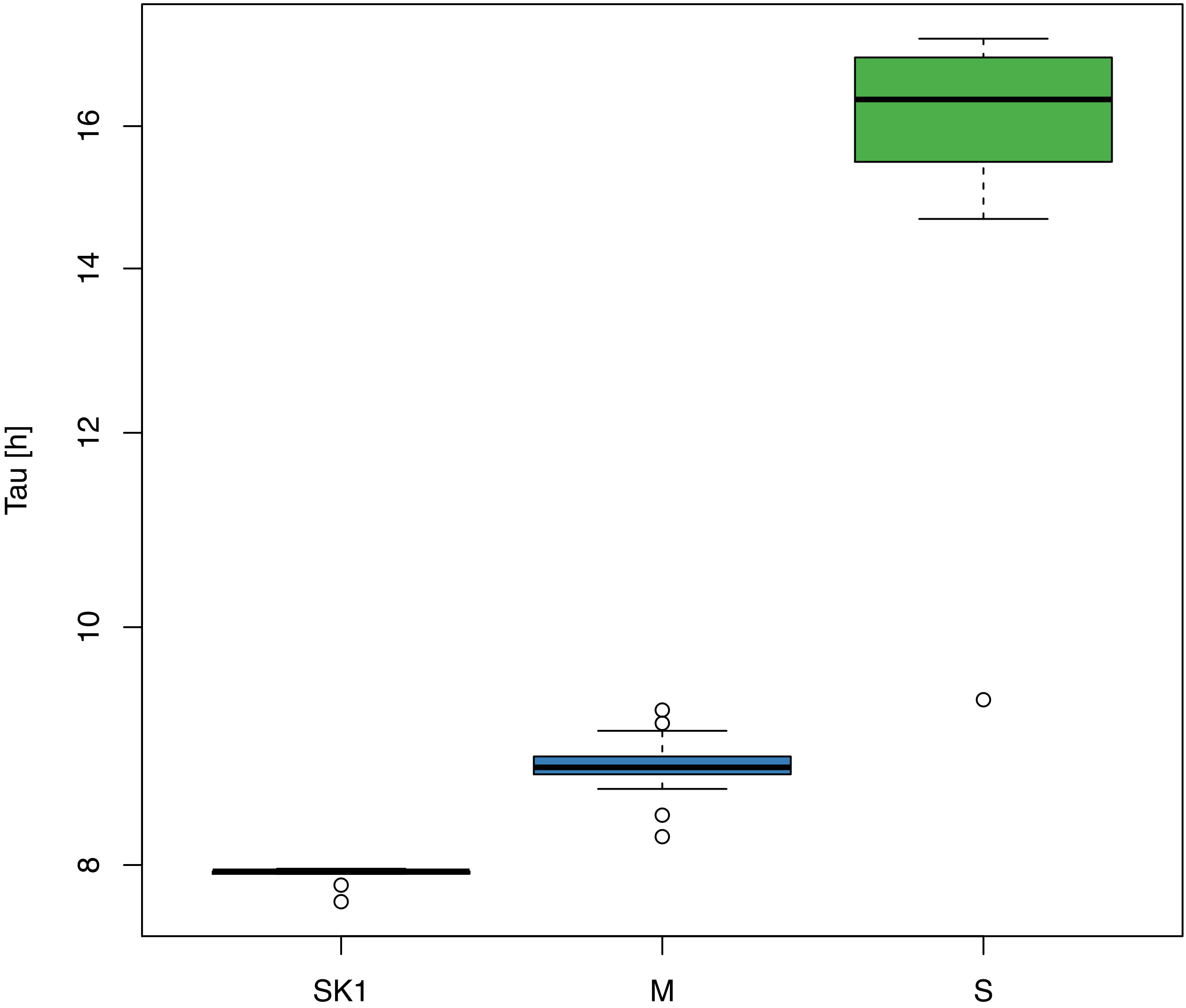

Supplement: S1 File — (ZIP) [file pgen.1005195.s022.zip › S1_File/S1_File.pdf]
